# Molecular dynamics in germinating, endophyte-colonized quinoa seeds

**DOI:** 10.1007/s11104-017-3184-2

**Published:** 2017-02-15

**Authors:** Andrea Pitzschke

**Affiliations:** Division of Plant Physiology, Department of Cell Biology, University of Salzburg, Hellbrunner Strasse 34, A-5020 Salzburg, Austria

**Keywords:** Quinoa, Bacterial endophytes, Germination, Mitogen-activated protein kinases (MAPKs), Elicitors

## Abstract

**Aims:**

The pseudo-cereal quinoa has an outstanding nutritional value. Seed germination is unusually fast, and plant tolerance to salt stress exceptionally high. Seemingly all seeds harbor bacterial endophytes. This work examines mitogen-activated protein kinase (MAPK) activities during early development. It evaluates possible contribution of endophytes to rapid germination and plant robustness.

**Methods:**

MAPK activities were monitored in water- and NaCl-imbibed seeds over a 4-h-period using an immunoblot-based approach. Cellulolytic and pectinolytic abilities of bacteria were assessed biochemically, and cellular movement, biofilm, elicitor and antimicrobial compound synthesis genes sequenced. *GyrA*-based, cultivation-independent studies provided first insight into endophyte diversity.

**Results:**

Quinoa seeds and seedlings exhibit remarkably complex and dynamic MAPK activity profiles. Depending on seed origin, variances exist in MAPK patterns and probably also in endophyte assemblages. Mucilage-degrading activities enable endophytes to colonize seed surfaces of a non-host species, chia, without apparent adverse effects.

**Conclusions:**

Owing to their motility, cell wall-loosening and elicitor-generating abilities, quinoa endophytes have the potential to drive cell expansion, move across cell walls, generate damage-associated molecular patterns and activate MAPKs in their host. Bacteria may thus facilitate rapid germination and confer a primed state directly upon seed rehydration. Transfer into non-native crops appears both desirable and feasible.

**Electronic supplementary material:**

The online version of this article (doi:10.1007/s11104-017-3184-2) contains supplementary material, which is available to authorized users.

## Introduction

Climate change-related land desertification and salinization increase at a worrying speed, driving the demand for novel crop cultivation concepts to ensure food security. Naturally drought- and salt-resistant plants have therefore experienced increasing attention in agriculture but also in the food industry. One prime candidate is the pseudo-cereal quinoa (*Chenopodium quinoa*), whose seeds have an outstanding nutritional value (Vega-Galvez et al. 2010). In its key agricultural countries - Peru, Bolivia, Argentina and Chile - quinoa experiences diverse harsh environmental conditions such as extreme aridity and frequent frost periods as well as salt concentrations that would be fatal to any other crop plant. In fact, some cultivars can complete their life cycle under sea water irrigation (app. 600 mM NaCl) (Panuccio et al. 2014). Because of its broad genetic variability in salinity tolerance quinoa is considered a valuable resource for selection of multiple stress-tolerant varieties and for breeding new varieties adapted to different environmental and geographical conditions (Biondi et al. 2016). Much like other halophytes, quinoa employs elaborate strategies to withstand osmotic challenges (reviewed in (Biondi et al. 2016)), such as decreased stomatal density/conductance, osmolyte accumulation and salt secretion via epidermal bladder cells (Orsini et al. 2011). Experts in the field believe that quinoa possesses additional, as yet unknown resistance mechanisms (Adolf et al. 2013), which might also account for quinoa’s robustness towards heavy metal stress (Pitzschke 2016). Quinoa overcomes one of the most critical stages in plant development, seed germination, very rapidly, with radicles protruding within less than one hour of imbibition. As proposed recently, seed-borne endophytes could be the “driving force” for cell expansion and resultant embryonal axis growth (Pitzschke 2016). Bacteria are apparently omnipresent in quinoa seeds, irrespective of batch, cultivar and origin. By generating (cell wall-loosening) superoxide and by providing enzyme activities (starch-mobilizing amylase) quinoa’s microbial partners potentially take an active share in host cell growth. “Seed bubbling” observed during the first hour of imbibition has been attributed to bacterial catalase-mediated oxygen production (Pitzschke 2016). All attempts to cure quinoa from its bacterial inhabitants have proven unsuccessful. Washes in ethanol did not remove them. (Pre)imbition in various antiobiotic solutions were –depending on duration and concentration - either inefficient or prohibited seed germination; the same applies to washes in bleach. Even heat-treated seeds (e.g. incubation 20 min at 80 °C) still contained endogenous, viable bacteria.

When placed on bacterial growth medium (YPD or LB agar), surface-sterilized aerial parts (cotelydons, stalks) of aseptically grown seedlings gave rise to colony growth, suggesting that bacteria can migrate *in planta.* Congruent with this assumption, bacterial suspensions showed high cell motility (Pitzschke 2016). Based on 16S rRNA gene sequences of colonies emerging from imbibed seeds (various batches tested), quinoa endophytes belong to the genus *Bacillus*; with several strains co-existing in a single seed (Pitzschke 2016). However, due to high percentage of 16S rRNA gene sequence similarity in *Bacillus* (Maughan and Van der Auwera 2011), more precise species affiliation requires sequence data of alternative marker genes such as *gyrA, cheA* (Reva et al. 2004).

Quinoa bacterial community members seemingly tolerate each other, and - as indicated by the lack of plant disease symptoms - they are also tolerated by the host (Pitzschke 2016). In this association, quinoa endophytes might put their host into a general alert state (induced resistance) (Pitzschke 2016). However, experimental support for this assumption has yet to be provided.

Virtually any plant species growing in free nature becomes inhabited by diverse microorganisms; roots are the primary entry sites (Partida-Martinez and Heil 2011). To harbor endophytic partners already before planting, i.e. at the dry seed stage, is less common, but not restricted to a specific phylogenetic lineage. Diverse endophytic bacteria have been found in seeds of e.g. eucalyptus (Ferreira et al. 2008), pumpkin (Furnkranz et al. 2012) and grapevine (Compant et al. 2011). While high cell motility and the ability to migrate into plants are properties shared by many endophytes, seed-borne endophytes rely on additional features to establish themselves inside seeds, a main prerequisite for trans-generational transfer via vertical transmission (Truyens et al. 2015). Endophytes secreting cell wall-degrading enzymes can use the nutrient-rich intercellular spaces of their hosts for migration. In contrast to endophytes colonizing plants at a later stage, seed-borne microorganisms must withstand high osmotic pressure, often over months or years. They must also be mobile in order to enter seeds before seed hardening, and readily resume their metabolic activities upon seed rehydration (Truyens et al. 2015). Hosts benefit from seed colonizers through e.g. improved seedling development, growth promotion and protection from pathogen attack (reviewed in (Truyens et al. 2015)). The benefit becomes even more evident under harsh environmental conditions: In its natural habitat, giant cactus grows on barren rock. Seed disinfection was found to prohibit seedling establishment, while plant development could be restored by inoculation with cactus endophytes (Puente et al. 2009). Endophyte composition analyses in five different bean cultivars revealed that seed-associated assemblages are primarily determined by soil type and humidity; not by the host genotype (Klaedtke et al. 2016). Accordingly, substrate composition turned out to be a decisive factor also for endophytic assemblages in Arabidopsis (Truyens et al. 2016b) and rice (Hardoim et al. 2012). Furthermore, from their observation that several members of the highly diverse endophytic communities from rice seeds overlap with those from the rhizosphere and surrounding soil (Hardoim et al. 2012) asked the intriguing question: “Are seed-borne endophytes selected by the host to increase the fitness of the next generations of seeds or do bacterial endophytes use seeds as vector for dissemination and colonization of new environments?” (Hardoim et al. 2012). These options need not be mutually exclusive. Barret et al. (2015) monitored bacterial and fungal community composition in 28 plant species (mostly Brassicacea) at three developmental stages (seeds; 24 h, 96 h post-imbibition) and found endophyte diversity to markedly decline during the transition to the seedling stage (96 h). The shift likely results from an increase in the relative abundance of bacterial and fungal taxa with fast-growing abilities (Barret et al. 2015). Johnston-Monje et al. compared four wild ancestors and ten varieties of modern maize in order to track endophyte assemblages during *Zea* domestication. Though endophytic bacteria identified by culturing, cloning and 16S rRNA gene-based classification substantially varied depending on host phylogeny, there was a core microbiota conserved across boundaries of evolution, ethnography and ecology. Selected genera were cultured and found to have growth-promoting, pathogen-antagonizing or other beneficial effects on treated plants (Johnston-Monje and Raizada 2011).

Certain molecular mechanisms governing developmental and stress responses are wide-spread among eukaryotes. Differences between species likely exist in the levels and kinetics at which these mechanisms are being activated. As evolutionarily conserved eukaryotic signalling modules, MAPK (mitogen-activated protein kinase) cascades play critical roles in the signalling of numerous developmental and stress adaptation processes. Cascade components are encoded by multigene families whose members have largely non-redundant functions. MAPK cascades amplify and transduce perceived environmental signals via a phosphorelay mechanism to effector proteins such as transcription factors (Choi et al. 2008). MAPKs act both up- and downstream of reactive oxygen species (ROS) (Pitzschke and Hirt 2009). Plant MAPK family members function as regulators of stomatal density/ stomatal aperture, mediate adaptation to drought, heavy metal, wounding, temperature stress and pathogen attack (Andreasson and Ellis 2010; Xu and Zhang 2015), and play a role in cell expansion (Sasabe and Machida 2012). Activity of the key Na+/H+ antiporter *At*SOS1, conferring ion homeostasis under alt stress, depends on MPK6 phosphorylation (Yu et al. 2010). Simultaneous loss of MPK3 and MPK6, causes defects in cell division, pollen development, stomatal distribution and stress adaptation (reviewed in (Xu and Zhang 2015) ). Arabidopsis MPK3-deficient plants are hypersensitive to salt stress and impaired in priming-induced resistance, as are mutants lacking MPK3 targets (Beckers et al. 2009; Greenberg et al. 2010; Kim et al. 2011; Persak and Pitzschke 2013; Pitzschke et al. 2014). Reciprocally, hyperactivation of MAPK signalling pathways may enhance resistance to various stress conditions, (Kim et al. 2011; Teige et al. 2004).

As part of the innate immune system, local contact of plants with elicitors, e.g. compounds on the surface of microorganisms, triggers multiple early responses, such as MAPK and antioxidant machinery induction, which provide a better and systemic protection against subsequent challenges (Andreasson and Ellis 2010; Pitzschke et al. 2009; Rasmussen et al. 2012). Because plants potentially recognize any microorganism, i.e. not only pathogens, as non-self, “harmless” microbes have potential to improve plant health, fitness and productivity (Wiesel et al. 2014) (Mueller and Sachs 2015). Owing to their diversity, microbial surface and secretion molecules may stimulate priming pathways in a very complex manner, improving plant resistance to various types of biotic and abiotic adversities (Reinhold-Hurek and Hurek 2011; Truyens et al. 2015).

Quinoa seeds that are directly sown in their field habitat, e.g. coastal zones, have to cope with high salinity stress right from the start, without any pre-adaptation period. Seedlings therefore rely on immediate mobilization of protective factors. Motivated by the recent discoveries on early developmental peculiarities and omnipresent seed-borne endophytes (Pitzschke 2016), the current work explores the hypothesis that by modulating host MAPK activities quinoa endophytes take an active share in rapid seed germination and plant stress resistance.

## Material and methods

### Plant material and treatment

To enable joint discussion with previous results, experimental conditions were basically identical to those described in (Pitzschke 2016). Quinoa seeds were sown into 12-well plates containing sterile tap water with or without 400 mM NaCl. Plates were incubated at 23 °C under moderate light. Per sample, 10–12 seed(ling)s, collected from separate wells, were snap-frozen in liquid nitrogen at the indicated time points and stored at −80 °C for protein analysis. Unless stated explicitly, seeds were type Real, harvested in Bolivia. Additional seed materials include: type Real (Peru), and varieties bred in Denmark: Puno, Titicaca and Vikinga; kindly provided by Sven-Erik Jacobsen (University of Copenhagen, Taastrup, DK).

### Protein extraction and immunoblot analysis

Protein extraction and immunoblot analyses were performed as described previously (Pitzschke et al. 2014). Frozen plant material was ground to a fine powder under liquid nitrogen using a bead mill (Retsch, Germany). Two volumes protein extraction buffer incl. Inhibitors (50 mM Tris/HCl pH 7.5, 5 mM EDTA pH 8, 5 mM EGTA pH 8, 2 mM DTT, 100 mM β-glycerophosphate, 10 mM Na-Vanadat, 10 mM Na-Fluorid, 10 mM PMSF, 10 μg/ml aprotinin, 10 μg/ml leupeptin) were added to the powder. Samples were thoroughly mixed and incubated 30 min on ice. The supernatant fluids obtained after centrifugation (15 min, 14,000 g, 4 °C) represent crude protein extracts. Protein concentrations in these extracts were determined using Bradford reagent and bovine serum albumin as standard. Protein concentrations were adjusted to 3 μg/μl by adding the respective amounts of extraction buffer. After sample denaturation in SDS-loading dye (added from freshly prepared 6-fold concentrate; final concentration 62.5 mM Tris/HCl pH 6.8, 2% SDS, 0.01% bromophenol blue, 10% glycerol, 100 mM DTT) for 5 min at 95 °C, 20 μg protein were separated by SDS–polyacrylamide gelelectrophoresis (Biorad minigel apparatus; 60 V, 150 min, room temperature). Proteins were subsequently transferred from the gel onto polyvinyliden difluoride (PVDF) membranes (Porablot, Roth) using a wet tank blotting apparatus (Biorad; 60 V, 80 min, 4 °C) and a pre-cooled transfer buffer (25 mM Tris, 192 mM glycine, 5% isopropanol). Membranes were blocked in Tris-buffered saline solution (TBS; 50 mM Tris/HCl pH 7.5, 150 mM NaCl) containing 1% soy protein (www.Sportnahrung.at) for 1 h at room temperature, and subsequently incubated over night at 4 °C in TBS-0.1% Tween (TBST) containing 1% soy protein and polyclonal rabbit antibodies at the following dilutions: Anti-ERK1p42/p44 (1:3000), anti-MPK3 (1:2000), anti-MPK4 (1:4000) and anti-MPK6 (1:4000).

Anti-ERK1p42/p44 antibody (CST signaling, UK), raised against the dually phosphorylated (p) peptide EHDHTGFLp**T**Ep**Y**VATR of mouse p38 MAPK, specifically recognizes active MAPK variants. Due to strong evolutionary conservation of this MAPK region and the phosphorylation-dependent activation anti-ERK1p42/p44 potentially recognizes any active MAPK in any eukaryotic organism (suppl. Fig. S1). Anti-MPK3, MPK4 and MPK6, which are directed against individual Arabidopsis MAPKs, recognize kinases irrespective of the activation state. More precisely, anti-MPK3 (Sigma) is directed against QEAIALNPTYG, anti-MPK4 (Davids Biotechnology) against MSAESCFGSSGDQS and anti-MPK6 (Davids Biotechnology) against FNPEYQQ.

**Fig. 1:**
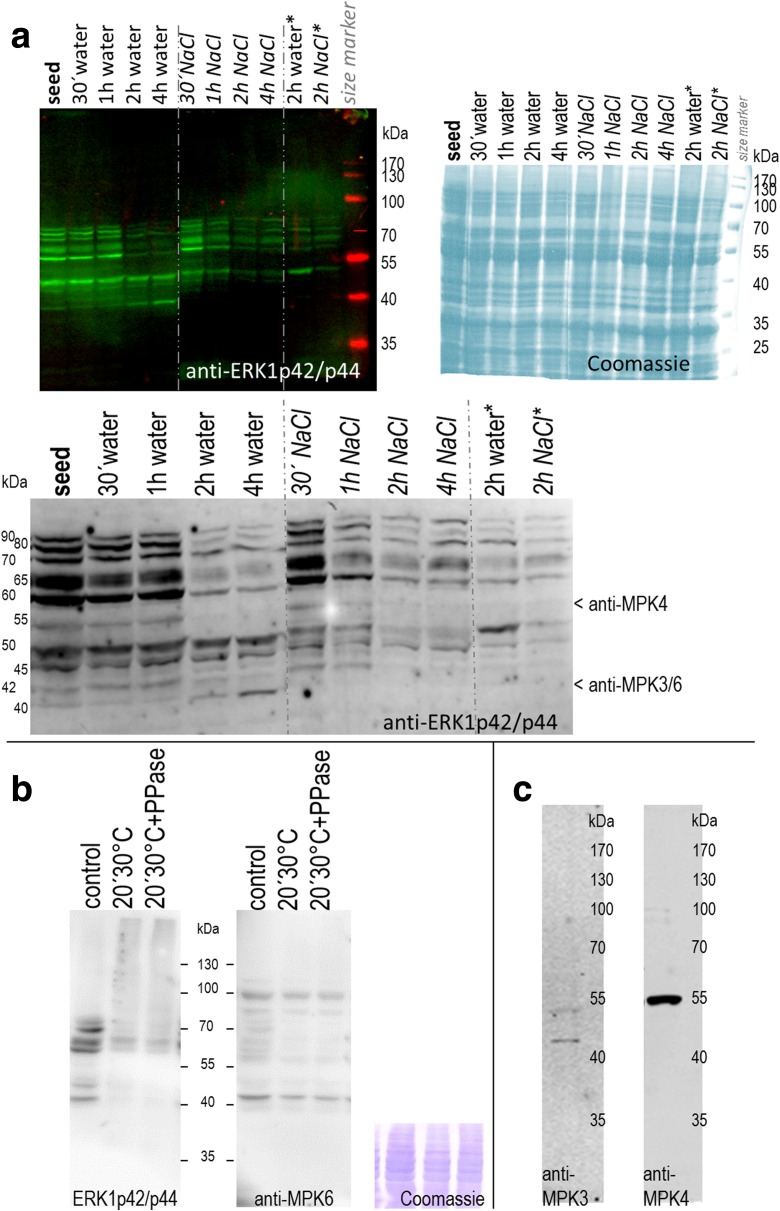
MAPKs in germinating and salinity-stressed seeds **a** MAPK activity profiles during germination and salt stress. Proteins (20 μg) were extracted from quinoa (Real_Bolivia) dry seeds and after imbibition in water or 400 mM NaCl. For the 2-h time point, extracts from an independent experiment were examined in parallel (columns at the right; marked with „*“). Active forms of MAPKs were detected by immunoblot analysis with anti-ERK1p42/p44 antibody and infrared dye-coupled secondary antibody. The blot was subsequently dried to enhance signal intensity, and a magnification of the 35–100 kDa region is shown in the lower part of the image. Arrows indicate protein bands potentially corresponding to anti-AtMPK3, −MPK4, and -MPK6 hybridisation signals (based on data of Fig. 1b/c). Right: A duplicate gel, containing the same samples, was stained with Coomassie blue to document protein loading. **b** Specificity of ERK1p42/p44 antibody. Quinoa seed protein extracts were incubated on ice (control) or at 30 °C for 20 min, without or with added phosphatase. Active forms of MAPKs were detected by immunoblot analysis with anti-ERK1p42/p44 antibody (left). The same membrane was subsequently hybridised with anti-MPK6 antibody (right), followed by CBB staining (bottom). Comparison of the two blots suggests that anti-ERK1p42/p44 signal reduction in treated samples (lanes 2&3) arises from dephosphorylation, not from protein degradation. **c** Cross-reactivity test with anti-Arabidopsis MAPK antibodies. Antibodies directed against evolutionary conserved peptides in Arabidopsis MPK3 and MPK4 were used for immunoblot analysis of quinoa protein extracts from (15 min)-water-imbibed seeds; 20 μg loaded)

**Fig. 2:**
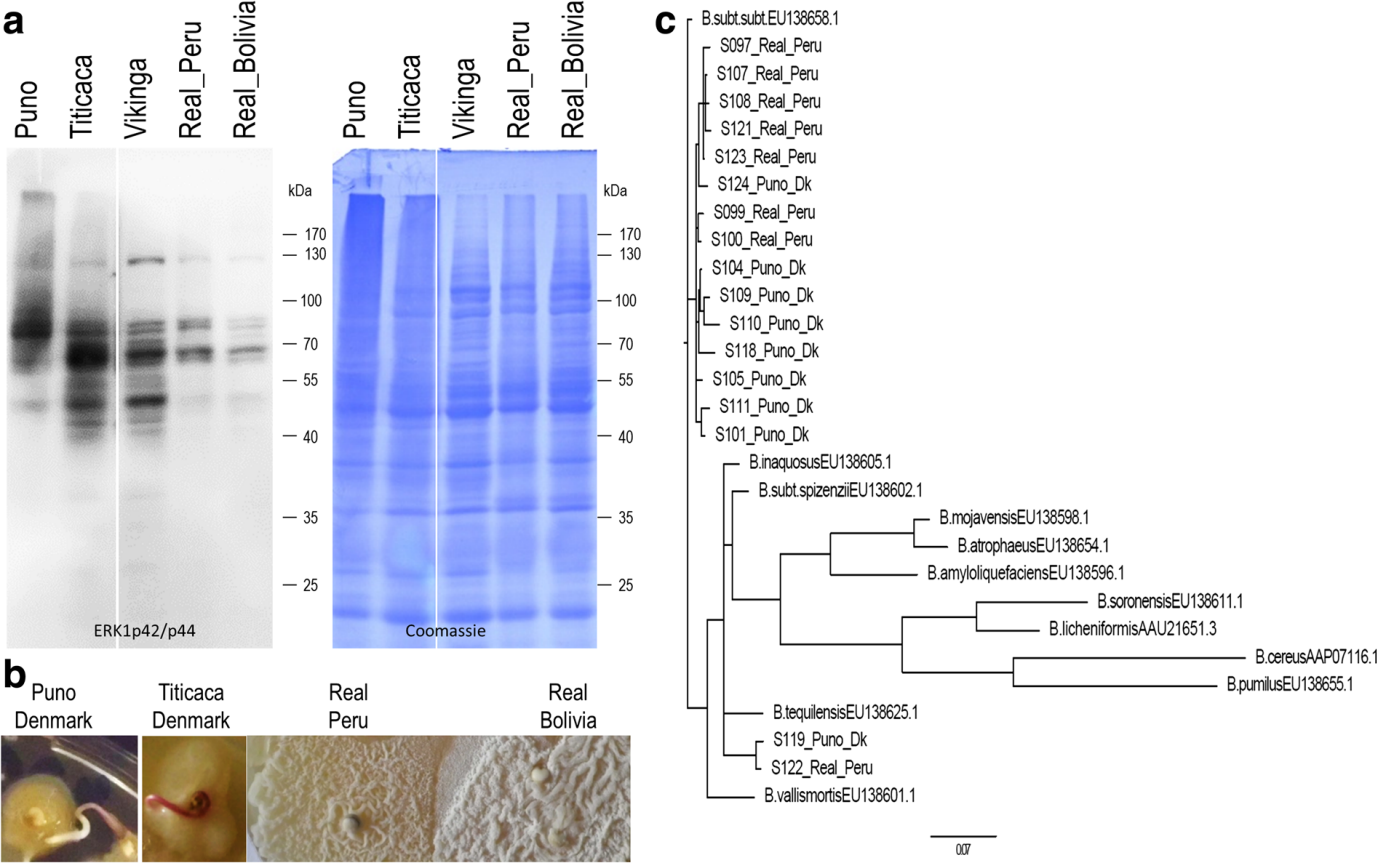
MAPK activity profiles and endophyte colonization in quinoa seeds from different sources. **a** Anti-ERK1p42/p44 immunoblot-based detection of MAPK activities in seed protein extracts. (Note: Differences in signal intensities (Real_Bolivia; Fig. 1 vs. fig. 2) are attributable to different secondary antibody detection systems). Coomassie blue staining served as loading control. **b** Imbibed seeds from the indicated sources were incubated on YPD agar, and emerging colonies fotographed after 4 days. **c** Divergence and phylogenetic relation of bacteria from two representative cultivars. The phylogenetic tree was constructed from bacterial *gyrA* genes amplified from metagenomic DNA of Puno (Denmark) and Real (Peru) seeds (see suppl. Data file for sequence alignment). Corresponding 685 nt *gyrA* sequences of *Bacillus* reference strains are included, and the tree was rooted using the *B. cereus* sequence

Membranes were washed in TBST (3 × 10 min), followed by incubation in secondary antibody solution (infrared dye-labelled anti-rabbit800CW (CST signalling, UK), diluted 1:20,000 in TBST/ 1% soy protein) for 1 h at room temperature. Membranes were washed 3 × 10 min in TBST and subsequently scanned according to the manufacturer’s instructions using the Odyssee® Infrared imaging system, at 800 nm for the detection of bound antibodies (Fig. 1). Alternatively, horse radish peroxidase (HRP)-conjugated anti-rabbit antibody (St. Cruz) was used as secondary antibody (diluted 1: 10,000 in TBST/ 1% soy protein), followed by chemiluminescence detection of H_2_O_2_/luminol substrate (Pierce ECL) conversion (Fig. 2a). Chemiluminescence signals were imaged according to the manufacturer’s instructions (LAS-3000 imaging system). Protein loading was documented by subsequent staining of PVDF membranes with Coomassie R-250 blue solution (0.05% Coomassie, 10% acetic acid). Experiments were repeated twice with similar results.

Tests on ERK1p42/p44 signal specificity followed a similar procedure, with some modifications: The buffer for seed protein extraction consisted of 50 mM Tris/HCl pH 7.5, 1 mM PMSF, 2 mM DTT and −/+ 100 mM ß-glycerophosphate. To facilitate dephosphorylation, two aliquots prepared without ß-glycerophosphate (a phosphatase inhibitor) were incubated at 30 °C for 20 min, in the presence or absence of exogenously added phosphatase (Lambda and calf intestine alkaline phosphatase; Fermentas). The control sample contained ß-glycerophosphate and was kept on ice. Anti-rabbit-HRP-conjugated antibody (St. Cruz), diluted 1:15,000 in TBST/ 1% milk, served as secondary antibody. After washing in TBST, membranes were incubated in H_2_O_2_/luminol substrate. And immunoreactive bands visualized by chemiluminescence detection (see above). Experiments were repeated twice with similar results.

### Bacteria sources

Bacteria used to examine swarming, CMC and pectin degradation as well as *flagellin*, *BmyD*, *PeBa1* sequences originated from quinoa seeds (type Real, Bolivia). They were either used as mixed cultures or as single strains; see (Pitzschke 2016) for 16S rRNA gene sequences.

### Assessment of swarming activities in quinoa endophytes

Bacteria proliferating on water-imbibed quinoa seeds, or from glycerol cultures of previously isolated candidates (Pitzschke 2016) were streaked on YPD medium (1% yeast extract, 2% bacto peptone, 2% glucose, 2% agar) and incubated at 25 °C for 2–5 days. Wrinkled colony formation was documented by photography.

### Cellulolytic and pectin-degrading activities in quinoa endophytes

Bacteria from freshly grown YPD plates were washed three times in 25 mM phosphate buffer (pH 7.0).

To assess cellulolytic activities, colony material was streaked with a toothpick on petri dishes containing 1.5% agar-solidified carboxy methyl cellulose (CMC) medium as used by (Singh et al. 2013) in a similar context (10 g/l CMC, 1 g/l K_2_HPO_4_, 1 g/l KH_2_PO_4_, 0.2 g/l MgSO_4_ *·*7H_2_O, 1 g/l NH_4_NO_3_, 0.05 g/l FeCl_3_ *·*6H_2_O (0.05), 0,02 g/l CaCl_2_). CMC utilization, visible as colony growth and surrounding clearing zones, was documented by photography.

To assess pectin-degrading activities, washed bacteria (see above) were diluted in sterile water (OD_600_ 0.2) and used as imbibition solution for *Salvia hispanica* (chia) seeds. Control samples consisted of chia seeds and water only. Tests were conducted at room temperature, in sterile 6-well-plates, and repeated twice with similar results. Pectin breakdown, visible as progressive disappearance of the mucilage seed coat, was documented by photography.

### PCR and sequence analysis of cultivated endophytes

Endophyte colony material was used as PCR template for amplification with primers for the *hag1* gene (flagellin) (Asano et al. 2001); *BmyD* (Xu et al. 2013); *PeBA1* (Wang et al. 2016)*, gyrA* (Reva et al. 2004). After PCR, using proof-reading polymerase and the following conditions: 95 °C 2 min, 35 x [95 °C 15 s, 50 °C 30 s, 70 °C 1 min], 70 °C 10 min, amplified products were separated on 1% TAE agarose gels, and selected samples sequenced (Microsynth, CH) using the respective forward primers. See suppl. Data for primer sequences.

### Sequences and alignments

Bacterial *flg, BmyD, PeBA1,* and *gyrA* as well as Arabidopsis MAPK sequences were retrieved from the NCBI database. Putative MAPKs from quinoa were identified by screening the quinoa genome database (http://quinoa.kazusa.or.jp/) for genes annotated as MAPKs (Yasui et al. 2016). Deduced protein sequences (Expasy Translation Tool) were aligned using the KALIGN (http://www.ebi.ac.uk/Tools/msa/kalign/) and MultAliN (http://multalin.toulouse.inra.fr/multalin/) tools.

### Cultivation-independent *gyrA* cloning and phylogenetic analysis

Ten surface-sterilized seeds of cultivars Puno (Denmark) and Real (Peru) were imbibed in water for two hours at room temperature and thoroughly washed in water. After seed maceration (using sterile metal balls and a bead mill) the material was processed for metagenomic DNA extraction (Maropola et al. 2015). 250 μl extraction buffer (25 mM Tris pH 7.5, 10 mM EDTA, 50 mM Glucose, 10 mg/ml lysozyme, 50 μg/ml RNAse) was added to each sample, mixed thoroughly and incubated for 1 h at 37 °C. Proteinase K was added (final concentration 1 mg/ml), followed by incubation at 37 °C for 1 h. SDS was added to a final concentration of 1%, and tubes were inverted ten times. After incubation at 65 °C for 30 min, samples were centrifuged at 14,000 g for 2 min, and the supernatants collected into new tubes. An equal volume of phenol was added and mixed by inversion. After centrifugation (10,000 g, 2 min) the upper aqueous phase containing DNA was collected into new tubes, and samples were extracted with phenol for a second time. An equal volume of chloroform/isoamyl alcohol solution (24:1; *v*/v) was added to each tube and mixed by inversion, followed by centrifugation (10,000 g, 15 min). DNA was precipitated from the upper aqueous phase by adding an equal volume of isopropanol and incubation at 4 °C for 30 min. Sample tubes were centrifuged (10,000 g, 5 min). Air-dried (RT, 10 min) DNA pellets were washed twice with 70% ethanol, (10,000 g, 5 min), dried again end re-suspended in 50 μl TE. 0.5 μl metagenomic DNA served as template in a 15 μl PCR reaction with primers *gyrA*_fo/re (suppl. Data file), proof-reading DNA-Polymerase and the following conditions: 95 °C 2 min, 35 x [95 °C 15 s, 50 °C 30 s, 70 °C 1 min], 70 °C 10 min. Amplification products were assessed by gel electrophoresis on 1% TAE-agarose gels. A-tailed PCR products were ligated into cloning vector pGemTeasy. *E. coli* transformants were screened by colony PCR (using pGem vector backbone-derived primers). Purified PCR products of 30 randomly selected clones (15 per cultivar) showing the expected insert size on TAE agarose gels were sequenced with primer *gyrA*_fo. Sequence alignments revealed that polymorphisms occurred over the entire gene region. With the intention to compare only non-ambiguous sequences, over a preferably long region, sequences with ambiguous residues, insufficient quality and/or length were discarded. The seventeen sequences remaining after manual editing and quality clips covered a 685 nt-long region in the *gyrA* gene. Respective regions of published *gyrA* sequences from *Bacillus* species reference strains (Rooney et al. 2009), retrieved from the NCBI database, were included in taxonomic classification studies.

Phylogenetic analyses employed the MrBayes software at http://www.phylogeny.fr/, with gamma distribution as setting for the likelihood model and the following Markov parameters: Generation of 100,000 trees, sampling every 100 generations, discard of the first 250 trees sampled. The resultant data in Newick format were processed for bootstrap analysis and tree construction with the http://tree.bio.ed.ac.uk/software/figtree/ software. *Bacillus cereus* served as outgroup to root the tree. Nucleotide sequences were deposited at NCBI Genbank.

## Results

Following quinoa seed imbibition in water or 400 mM NaCl, radicle protrusion occurred within 30 min. Consistent with previous observations there were bubbles steadily arising from seed surfaces, indicative of catalase-mediated oxygen production (Pitzschke 2016). While not affecting radicle protrusion and early development (first few hours), 400 mM NaCl did slow down subsequent development (cotelydon emergence; root growth). As noted by (Adolf et al. 2013) already, salinity primarily delays germination commencement before affecting the germination percentage. In an attempt to see how rapid seedling development manifests itself at the molecular level, subsequent experiments focussed on MAPKs.

### MAPK activity profiling

Owing to the high conservation of eukaryotic MAPKs, experimental tools developed in animal science can also be used in plant research (reviewed recently by (Pitzschke 2015; Xu and Zhang 2015)). In particular, “ERK1p42/p44” antibodies targeting a universal, conserved motif in active variants of mammalian MAPKs (suppl. Fig. S1), have proven valuable for the detection of MAPK activities in plant extracts. These antibodies were therefore also deemed suitable for monitoring MAPK activities in quinoa (see below; suppl. Fig. S2). A central question was whether the rapid physiological changes (i.e. radicle protrusion, embryo expansion) would be accompanied by similarly rapid changes in individual MAPK activities, and whether stress-related responses could potentially be signalled via MAPKs.

Following imbibition in tap water or 400 mM NaCl for a period of 30 min, 1, 2 and 4 h, seeds were processed for protein extraction and MAPK analyses. As evidenced by Coomassie blue staining of extracts separated by SDS-PAGE the overall protein patterns in all samples largely overlapped (Fig. 1). Development- and/or treatment-specific differences in protein profiles may become evident later on, as e.g. shown in a recent report on adult plants (Aloisi et al. 2016). Hybridisation signals obtained with anti- ERK1p42/p44 antibody, specifically recognizing *activated* dually phosphorylated MAPK variants suggest presence of at least ten putative MAPKs, which are simultaneously active in quinoa seed(ling)s (Fig. 1) – an astonishingly high pattern complexity compared to similar materials from other plant species (Barba-Espin et al. 2011; Brock et al. 2010; Testerink et al. 2000). Protein sizes (app. 40–90 kDa) were in the typical range of plant MAPKs reported so far (22–98 kDa) (Mohanta et al. 2015). Hybridisation signals of two protein bands (app. 60 and 65 kDa) dominated, pointing to particularly strong activities of the respective kinases. Overall, MAPK activities were highest in seeds and decreased during germination. Only one MAPK, with an estimated size of 40 kDa, showed higher activities in fully rehydrated as compared to dry seeds. Changes in MAPK activities occurred soon after imbibition. Under non-stressed conditions, these non-linear changes were most pronounced between the 1-h and 2-h time point. In contrast, under high-salinity conditions the most prominent differences appeared already between the 30-min and 1-h time point. Individual MAPKs can be further classified according to their activation kinetics:

#### Progressive inactivation

For both treatments, kinase activities at approximately 80, 60, 55, 50 and 42 kDa changed in a monophasic manner, and activity loss was accelerated in the presence of salt (Fig. 1a). For instance, imbibition in salt solution caused a sharp decline in signal intensity at 50 kDa, which decreased further (1 h) to fall below the detection limit (2 h, 4 h). Though these signals did progressively disappear also in water-imbibed seeds, band intensities were still stronger at 4 h post-imbibition, as compared to any NaCl-treated sample.

#### Treatment-dependent re-activation

Seed imbibition in water triggered a pronounced decrease in approximately 70 kDa-sized MAPK activities between the first end second hour; and levels remained constant later on (Fig. 1a). This drop in activity occurred earlier (between 30 min and 1 h) and was transient in salt-imbibed seeds. Here, kinase activities at 4 h post-imbibition had returned to levels comparable to the 30-min time point. The situation was reverse for an app. 42 kDa MAPK, where activities in salt-imbibed seeds decreased early (30 min) and remained low thereafter. Water-imbibed seeds, however, showed a transient drop (30 min, 1 h), followed by a gradual increase (2 h, 4 h).

#### Overlapping profiles

Interestingly, for salt-imbibed seeds, signal intensities at 90 kDa and 70 kDa in a given sample were similar and changed in an identical manner during the entire four-hour observation period.

### Specificity of ERKp42/p44 signals

To attain assurance that the signals indeed corresponded to MAPK activities, protein samples were treated in vitro prior to immunoblot analysis. Seed proteins extracts were prepared in the presence or absence of exogenous phosphatase inhibitors. The control sample, containing beta-glycerophosphate (GLP) as a serine/threonine phosphatase inhibitor, was kept on ice throughout. Two samples, lacking GLP, were incubated at 30 °C for 20 min. One of these contained recombinant phosphatase. The reasoning was that endogenous and/or exogenous phosphatases would convert active MAPKs into their inactive form, resulting in loss of the ERKp42/p44-specific epitope. Indeed, compared to the control sample, moderate sample warming lead to substantial reduction of signal intensities (Fig. 1). Irrespective of phosphatase addition, both 30 °C-incubated samples showed basically identical and comparatively weak signal profiles. From these observations one may conclude that i) the antibody-based approach was appropriate for visualising *active* forms of MAPKs and that ii) quinoa contains endogenous phosphatases able to effectively deactivate MAPKs.

### Immunoblot-based detection of Arabidopsis MAPK orthologs in quinoa

Among the 20 MAPKs encoded by the *Arabidopsis thaliana* genome three representatives - MPK3, MPK4 and MPK6 – have attracted particular attention in developmental and stress research (Droillard et al. 2004; Pitzschke 2015; Rasmussen et al. 2012; Xu and Zhang 2015). Antibodies targeting evolutionary conserved patterns in these kinases are also functional in species outside the Brassicacea (Li et al. 2007). In contrast to anti-ERK1p42/p44 (see above) recognizing active kinase variants only, these antibodies function irrespective of their target’s activation state. With the intention to assign ERK1p42/p44 hybridisation signals (Fig. 1a) to putative Arabidopsis MAPK orthologs, quinoa protein extracts were examined by immunoblotting with anti-MPK3, MPK4 and MPK6 antibodies. Both anti-MPK3 (Fig. 1c) and anti-MPK6 (Fig. 1b) reacted with proteins of an approximate molecular weight of 42 kDa; a size similar to *Arabidopsis* MPK3 (43 kDa) and MPK6 (45 kDa). Anti-MPK4 generated a single strong immunoreactive band with an apparent Mw of 55 kDa (Fig. 1c), appreciably larger than Arabidopsis MPK4 (43 kDa) (see below). Notably, MAPK activities at around 55 kDa can be seen on the anti-ERK1p42/p44 blot (Fig. 1a). MAPK-activity signals around 42 kDa, potentially corresponding to MPK3 and MPK6, are comparatively weak. Their intensities change in a stimulus- and time-dependent manner.

### Seed source-dependent microbial communities and MAPK profiles

As it was found impossible to cure quinoa from its endophytes (see introduction), quinoa can currently be studied only as holobiont. Consequently, it is anything but trivial to unequivocally link MAPK activities to the presence of (particular) bacterial factors or functions. The fact that multiple strains *co*-exist in seeds complicates things further. To still shed light on this issue, MAPK activity profiles were examined in seeds from quinoa type Real, cultivated in Peru and Bolivia, and in three Danish-bred varieties. Immunoblot analyses with anti-ERK1p42/p44 unveiled differences in the patterns of MAPK activities, depending on seed origin (Fig. 2a). When plated on solid YPD medium, seeds from the different sources gave rise to morphologically different colonies (Fig. 2b). All bacterial materials tested were found catalase positive, and microscopy analyses revealed high cell motility, sporulation-competence and the tendency to form chain-like aggregates– typical characteristics of *Bacilli*. From this one cannot judge yet on the degree of diversity because especially *Bacilli* are well known for having different morphotypes within a strain. Next experiments therefore involved comparative DNA sequence analyses. In *Bacillus* the 16S rRNA gene contains insufficient phylogenetic information for resolving closely related members (Rooney et al. 2009). Therefore, *gyrA*, a polymorphic marker suitable for taxonomic analyses on *Bacillus* (Oslizlo et al. 2015; Rooney et al. 2009) was employed here. In order to avoid any potential bias caused by cultivation, metagenomic DNA was extracted from seeds directly, i.e. without cultivation on YPD or other artificial medium. Two cultivars, Puno from Denmark and Real from Peru, were selected as representatives of different origin and clearly distinct MAPK activity profiles. *gyrA* sequences of randomly chosen *E. coli* colonies obtained after *gyrA* PCR amplification and cloning were determined. Sequence alignment (**suppl. Fig. S** **3**) revealed several polymorphisms between individual sequences, within and between the two cultivars. Noteworthy, all seventeen sequences inspected are distinct from each other. The majority (15 sequences) can all be assigned to *Bacillus subtilis* subsp. *subtilis;* and two (S119 Puno_Dk and S122 Real_Peru) are more closely related to *B. tequilensis/ B. inaquosus* (Fig. 2c). Interestingly, sequences derived from Puno_Dk or Real_Peru fall into different subclades on the phylogenetic tree; with S124_Puno being the only exception. Sequence diversity among endophytes from Puno_Dk appears to be lower as compared to Real_Peru. That isolates from Puno vs. Real tend to cluster might point to the existence of cultivar/origin-specific bacterial assemblages, though for a clear answer substantially more sequence data are needed.

### In silico studies on quinoa MAPKs

**Fig. 3:**
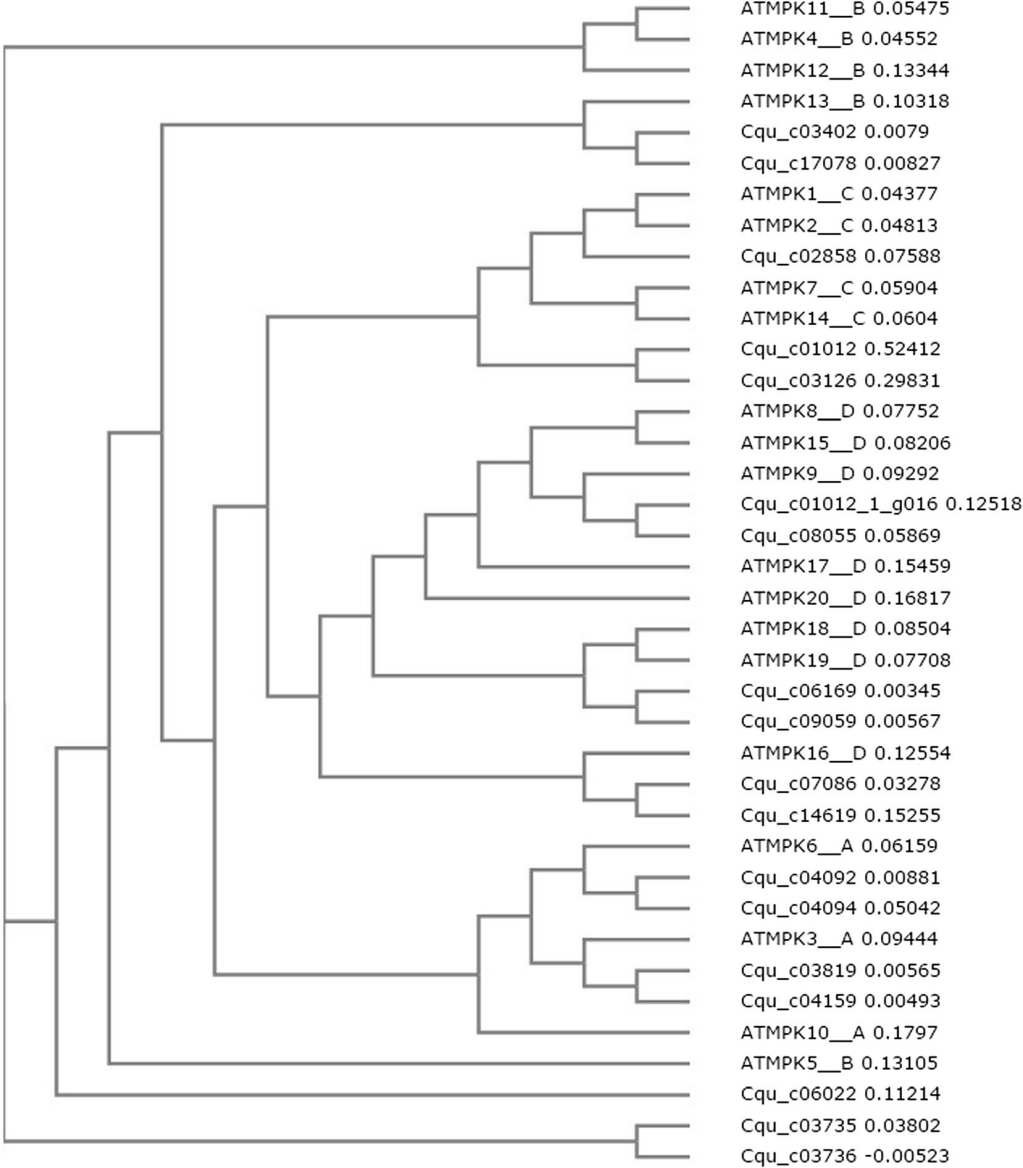
Phylogenetic relation of the 18 putative quinoa MAPKs with all 20 Arabidopsis MAPKs. For Arabidopsis MAPKs the respective subgroup (**a**,**b**,**c** or **d**) is indicated. The tree was generated from amino acid sequence alignments (suppl. Fig. S2)

Until recently quinoa DNA sequence information had been very limited, and MAPKs totally unexplored. None of the BAC clone sequences (Stevens et al. 2006), or the 424 EST entries in the NCBI database (Coles et al. 2005) displays convincing homology to MAPKs (concluded from BLASTN searches). During preparation of this manuscript (Yasui et al. 2016) reported on the draft genome sequence of an inbred quinoa line. The tools and data generated thus enable first predictions on quinoa MAPK family sizes and phylogenetic relations. From screening of the quinoa genome database (QGDB) eighteen genes annotated as MAPKs could be identified. Subsequent amino acid sequence alignments with the members from Arabidopsis (Fig. 3 and suppl. Fig. S2) disclosed substantial homology, emphasizing the evolutionary conservation of MAPKs. The MAPK protein family consists of four groups, A, B, C and D, characterized by distinctive features in their primary protein structures (Ichimura et al. 2002). Judged from in silico analyses, i.e. sequence alignments (suppl. Fig. S2) and phylogenetic evaluation (Fig. 3) quinoa contains MAPK representatives in all four groups. From the phylogenetic tree (Fig. 3) existence and identity of a quinoa MPK4 homolog is not evident. The strong 55 kDa-sized hybridization signals obtained with anti-AtMPK4 antibody likely derives from Cqu_c04092.1_g025.1, whose calculated molecular weight is 54.1 kDa. Its amino acid sequence contains a motif (PPENHPPP**SSDQS)** resembling the anti-MPK4 epitope MSAESCFG**SS**G**DQS**. Notably, the motif lies outside the conserved kinase domain (suppl. Figs. S2), and other putative quinoa MAPKs lack such or similar motifs.

### Putative elicitors derived from quinoa endophytes

To my knowledge, MAPK activity profiles as sophisticated as in quinoa have not yet been observed in any other plant, raising the suspicion that they arise from some distinguishing features not commonly found in the plant kingdom. I considered factors or functions that originate from quinoa’s bacterial inhabitants as prime suspects. Subsequent experiments therefore focussed on bacterial factors and functions that potentially contribute to host MAPK activation.

**Fig. 4:**
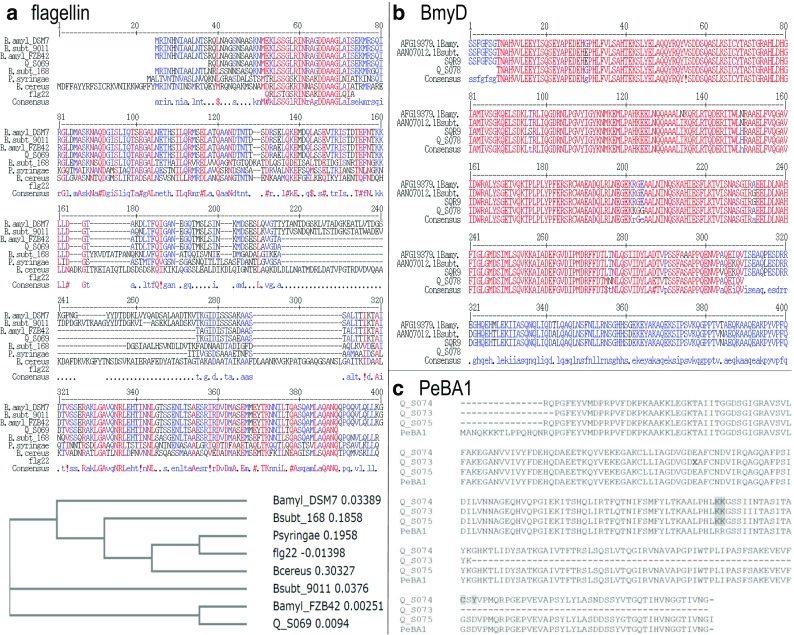
Putative elicitors, antimicrobial and biofilm compounds in quinoa endophytes**. a** Alignment and phylogenetic relations of amino acid sequences from quinoa endophytes with flagellin sequences from *B. amyloliquefaciens, B. subtilis, B. cereus*, and pathogenic bacteria (*Pseudomonas syringae*). Flg22, a commonly used elicitor peptide, is included in the alignment. Accession numbers: B.amyl_DSM7: WP_013353776.1, B.amyl_FZB42: ABS75581.1, B.cereus: AAZ22698.1, B.subt_168: CAB15553.1, B.subt_9011: BAB58972.1, P.syringae: WP_046719188.1. **b** Alignment of bacillomycinD synthetase (partial) sequences from *Bacillus* strains exhibiting antifungal and biofilm-forming activities with putative homologs from quinoa endophytic bacteria. **c** Alignment of the induced systemic resistance (ISR)-triggering elicitor PeBA1 from *B. amyloliquefaciens* with (partial sequences of) putative homologs from quinoa endophytic bacteria, deduced from sequenced PCR products of three independent strains. Positions differentiating from the PeBA1 sequence are highlighted

Peptides derived from flagellin, a component of bacterial flagella, are well-known MAPK elicitors (Pitzschke et al. 2009). Though the high cell motility exhibited by quinoa endophytic bacteria (Pitzschke 2016) indicates flagellar movement, sufficient evidence for the existence of flagellin is still lacking. To remove this uncertainty, flagellin-coding genes (*hag*) were amplified from bacteria and their sequence determined. Own preliminary examination involving sequence alignments of previously reported flagellin primer sequences (Asano et al. 2001) with the respective genes from diverse bacteria revealed sufficiently high conservation, suggesting a more general/universal suitability of these primers. PCR amplification yielded an approximately 1 kb-sized product in all of four independent strains tested. Product identity was confirmed by sequencing. As seen in the alignment of the deduced amino acid sequences with the respective peptides from other bacteria (Fig. 4a) homology is particularly strong to flagellin from DB9011. *Bacillus* strains with antifungal activities reportedly form a distinct group, recognizable by specific residues in their flagellin sequence (Asano et al. 2001). Notably, the quinoa endophyte-derived sequence falls into that group.

**Fig. 5:**
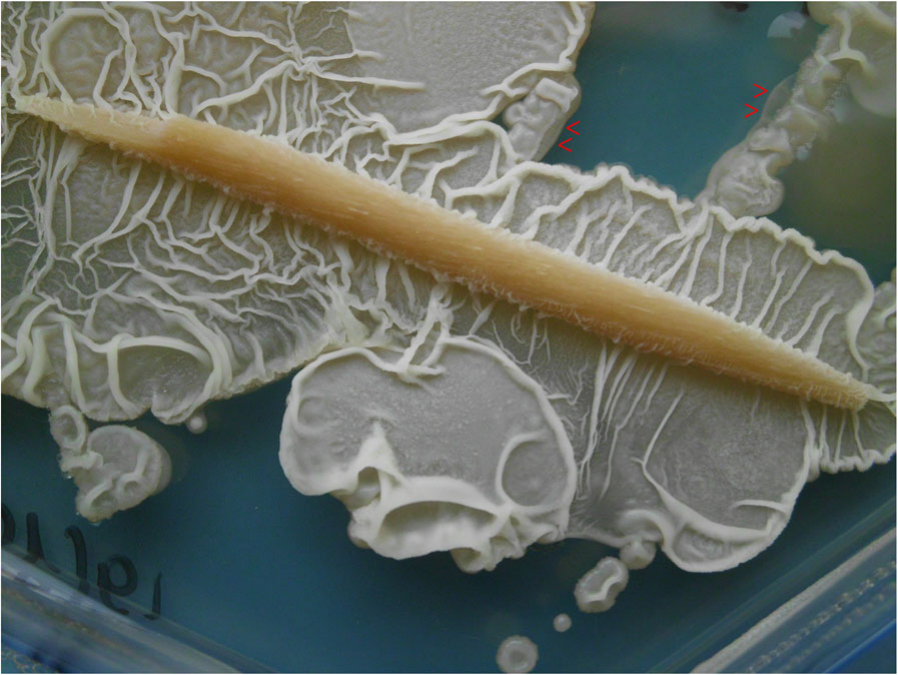
Swarming behaviour and biofilm formation. When incubated on solid surfaces, quinoa endophytes form wrinkled colonies. Note the watery layer preceding the cells at the swarm front (*red arrows*). The foto shows colony expansion on YPD agar onto which a wooden toothpick had been placed prior to inoculation

Besides flagellin, various biofilm components, can act as elicitors to induce systemic resistance (Ongena et al. 2007). Flagellin-driven swarming and biofilm formation are frequently found in *Bacillus*, and wrinkled colony structures considered a hallmark of biofilm production (Vlamakis et al. 2013). In that respect quinoa endophytes behave very similar, as evidenced by formation of wrinkled colonies upon bacterial cultivation on solid YPD medium (Fig. 5). At the expansion front, colonies had a shiny, moist appearance and smooth surface (Fig. 5, arrows), indicative of biofilm component secretion. Because identification and characterisation of secretion products would exceed the scope of this manuscript, I aimed to at least test whether quinoa endophytic bacteria possess critical genes (*BmyD* and *PeBa1*) associated with elicitor biosynthesis. Bacillomycin D is a lipopeptide, previously isolated from secretions of *B. amyloliquefaciens* SQR9. It derives from a screen for pathogen-suppressing compounds of growth-promoting bacteria. Bacillomycin D is a major antibiotic against the soil-borne fungal pathogen *Fusarium oxysporum*, and it was also found indispensable for biofilm formation (Xu et al. 2013). Colony PCR using *BmyD* primers yielded PCR products in all of four samples tested, and DNA sequencing confirmed product identity. The alignment (Fig. 4b) includes sequences of two additional *Bacillus* strains whose antifungal properties and potential as biocontrol agents are well-documented (Moyne et al. 2004; Zhao et al. 2014).

#### PeBA1

PeBA1, isolated from *Bacillus amyloliquefaciens* NC6, has recently been reported as potent inducer of systemic resistance in tobacco (Wang et al. 2016). Mass spectrometry enabled peptide sequencing and subsequent cloning of the *PeBA1* gene for recombinant protein expression and purification (Wang et al. 2016). Published *PeBA1* primer sequences served here for testing presence of *PeBA1*-like genes in quinoa endophytes. PCR products could be amplified from all four samples tested. The deduced amino acid sequences slightly vary between individual colonies, but all exhibit high homology to PeBA1 from *B. amyloliquefaciens* NC6 (Fig. 4c).

PCR amplification with *flagellin, BmyD* and *PeBA1*, as well as with *CheA* and *gyrA*-specific primers also yielded products of the expected sizes when using imbibed seeds or bacterial material (no single colonies) as templates. However, no sequence information could be retrieved from these samples due to chromatogram heterogeneity. This observation emphasizes the notion of community complexity.

#### Cell wall degrading/loosing activities: DAMPs and host cell growth

DAMPs (damage-associated molecular patterns) arising e.g. from wounding or from microbial enzyme-mediated cleavage of host structures knowingly induce MAPK pathways (Boller and Felix 2009; Heil and Land 2014), and cell walls present rich sources of potent MAPK activators, such as polysaccharides (Holley et al. 2003) and pectin fragments (Nuhse et al. 2000) (reviewed in (Ferrari et al. 2013). Against this background, quinoa endophytes were tested for their ability to break down cellulose and pectin. Because cell wall-loosening is mandatory for cell expansion, the experiments also addressed possible contributions of bacterial enzyme activities to host growth. Such activities would furthermore assist bacterial migration *in planta*, i.e. movement across cell barriers.

#### Cellulose breakdown

**Fig. 6:**
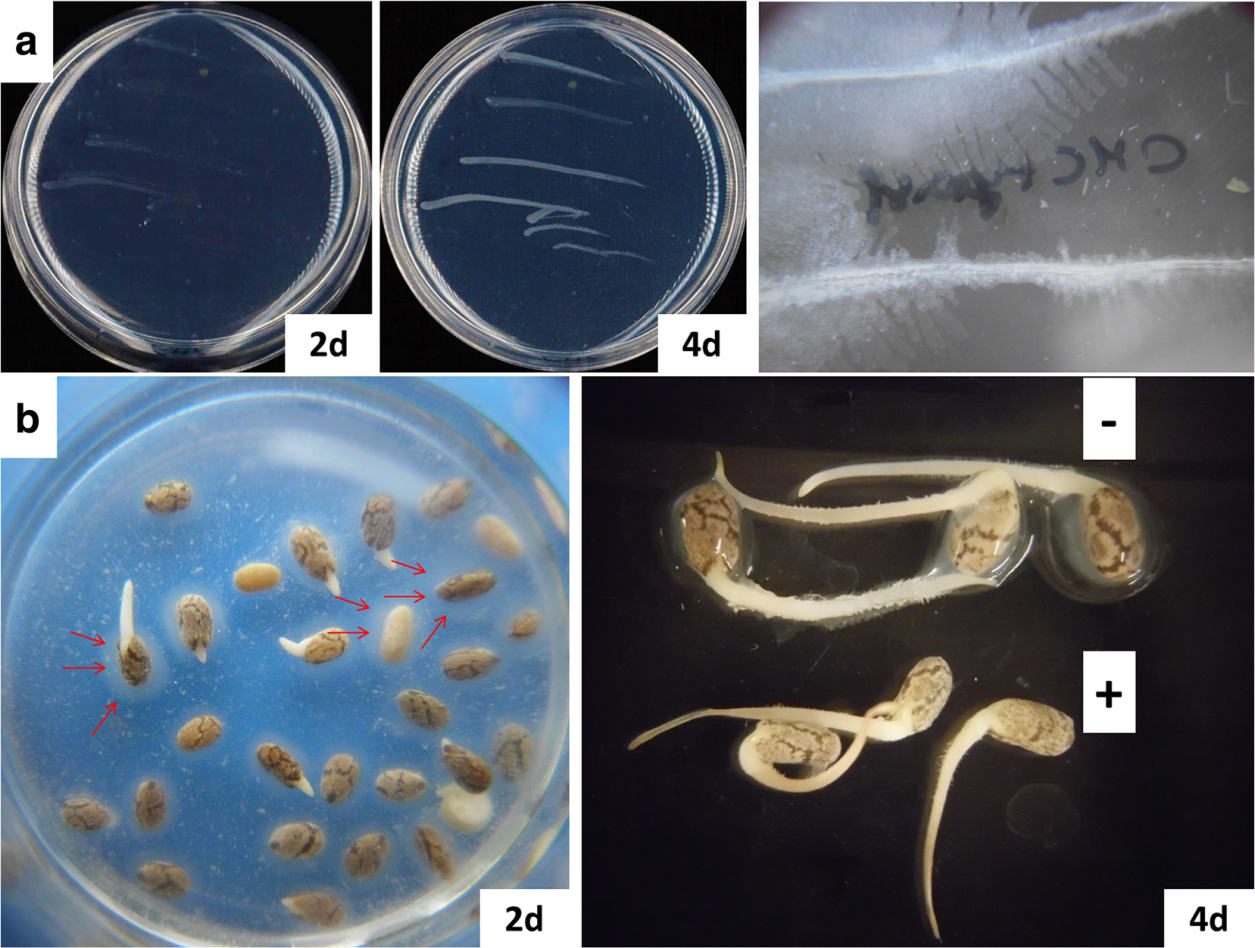
Cellulolytic and pectinolytic activities of quinoa endophytic bacteria. **a** Four independent colonies were re-streaked on CMC medium and incubated at 25 °C. Right: On CMC medium that had been supplemented with yeast the horizontally streaked colonies ( 2 streaks shown) expanded to form brush-like structures. **b** Bacterial suspensions in sterile tap water were used for chia seed imbibition. Left: Arrows indicate the mucilage coat, which was progressively degraded. Right: Seedlings four days after imbibition in water (−) or bacterial suspensions (+)

Colony materials from single strains, corresponding to previously published 16S rRNA gene sequences, as well as a mixture of randomly chosen colonies were streaked on carboxymethyl cellulose (CMC) medium. Bacterial growth became evident after app. 40 h of incubation. By day 4, all samples had formed substantial amounts of cell material (Fig. 6a). Importantly, the medium lacked any additional carbon source, and only contained an inorganic nitrogen source (NH_4_NO_3_). These findings indicate that quinoa endophytes are not only capable of cellulose degradation, but they can also use cellulose as the sole carbon and energy source.

#### Pectin breakdown

Pectins form a group of chemically diverse heteropolysaccharides found in the primary cell walls of terrestrial plants. Their industrial production, primarily from citrus and apple peel, involves heating in hydrochloric acid, precipitation with ethanol, and heat-drying. Such harsh isolation procedures inevitably affect pectin chemistry and structure. Given this background, native pectin sources appeared more appropriate for assessing cleavage capabilities of quinoa endophytes.

Chia seeds offer a convenient source of pectin-like substances. Upon contact with water, seeds exudate mucilage material that sticks to the seed surface, visible to the naked eye. The gelling component, characterized by a high water retention capacity, is a polysaccharide consisting of partially methylated glucurono- and xylopyranosyl derivatives (Lin et al. 1994). Chia mucilage furthermore contains proteins and fibers (Capitani et al. 2013). To test whether quinoa endophytes can degrade mucilage materials, chia seeds were imbibed in water or in bacterial suspensions, respectively. After approximately 20 min all seeds had formed mucilage coats of similar thickness (app. 2 mm). In the case of water-imbibed seeds they were still apparent after four days of incubation at room temperature. However, seed imbibition in bacterial suspension lead to progressive loss of mucilage material, documented at day four (Fig. 6b). Experiments with chia seeds that had been pre-treated (30 min 99 °C to inactivate any endogenous enzymes) prior to imbibition supported the notion that mucilage degradation was attributable to quinoa-derived bacterial activities. Heat treatment blocked chia seed germination, but mucilage production and cleavage were basically identical to that in non-heated seeds.

## Discussion

### MAPK profiles

Molecular principles underlying the exceptional stress robustness and rapid germination in quinoa are poorly understood. This study on quinoa seed(ling)s revealed an unusually high complexity and dynamics in MAPK activities. Experiments performed under dephosphorylation-facilitating or –blocking conditions provided further evidence that immunoblot hybridisation signals truly derived from active MAPK variants. Three of those could be assigned to putative homologs in Arabidopsis. The decline in several MAPK activities was faster in salt- as compared to water-treated quinoa, suggesting that salinity promotes the germination-related cellular reprogramming. Such loss of MAPK activities may result from i) progressive induction of germination-responsive MAPK-specific phosphatases, as e.g. known from Arabidopsis PP2C5 (Brock et al. 2010); ii) germination-specific proteolytic MAPK degradation; or iii) inactivation of the upstream regulatory MAPK kinase. Within the comparatively short observation period, i.e. 4 h, most MAPKs displayed time- and treatment-specific changes in their activities. MAPK activity profiles in water vs. salt-treated quinoa diverged early upon imbibition and became more similar again (4 h). A short period of markedly differential activities (30 min-2 h) appears to be sufficient for triggering appropriate long term adaptation responses. Such timing can diminish the risks of excessive or prolonged, or even potentially non-specific, target protein modification.

With one exception only, there was no complete signal profile overlap between any two representatives (i.e. strictly identical (in)activation pattern over the four-hour period in water- and salt-imbibed seeds), indicating largely non-redundant functions of the ten MAPKs monitored. Profile identity of a70 kDa- and 90 kDA-sized band could be attributable to co-regulation by a common upstream MAPK kinase. Similar scenarios are known from *Arabidopsis* MAPK3 and MAPK6, which have partially non-redundant functions in plant development, but are co-regulated by MAPKK4 under stress (Pitzschke 2015).

Well-tuned molecular adjustments in MAPK activities seemingly occur during early embryo development, a phenomenon that - to my knowledge – has not been reported for any other species so far. For instance, an immunological approach revealed presence of three MAPK proteins, sized 30, 45 and 60 kDa, in barley grains (Testerink et al. 2000). Only the 45 kDa MAPK displayed detectable activities which decreased during the first day of imbibition. This would somewhat parallel, “in slow-motion”, the overall decline in MAPK activities found in germinating quinoa. Future work involving gel excision and subsequent mass spectrometry should allow assignment of MAPK activation signals to individual MAPKs; a task facilitated by availability of a quinoa draft genome sequence (Yasui et al. 2016). It will also be interesting to compare MAPK family divergence between different cultivars.

### Endophytes as MAPK-cascade stimulators

All quinoa seeds harbour endophytic bacteria. The seemingly strong interdependence (failure to remove bacteria without killing the host) makes it currently impossible to provide final proof for bacterial (co-)responsibility for quinoa’s physiological and molecular (MAPK) peculiarities. However, the data (Figs. 1 and 2) strongly point into that direction. It appears reasonable to assume that individual strains contribute differently to host MAPK activation, yielding a net effect of the entire community on host responses. Variances in community composition would therefore be expected to entail distinct MAPK profiles. Indeed, depending on seed origin, differences in MAPK activity profiles (Fig. 2a), and seemingly also in microbial community composition were recognizable. In line with the notion that soil composition/humidity, is a key driver of microbial assemblages (Klaedtke et al. 2016; Truyens et al. 2016b), colony morphologies differed between bacteria arising from quinoa type Real seeds from Peru vs. Bolivia; and they were also distinct from Danish-bred varieties (Fig. 2b). *GyrA* sequencing of randomly selected clones provided first insight into the complexity and divergence in endophyte communities of two selected cultivars. Resembling the situation in tomato rhizoplane (Oslizlo et al. 2015), most candidates belong to *Bacillus subt.* Subsp. *subtilis* but other *Bacilli* species are also represented (Fig. 2c). Future NGS analyses shall enable identification of the entire microbiomes; a prerequisite for assignment of bacterial assemblages to MAPK activity profiles. In several plant species, individual MAPKs have been ascribed to specific stress adaptation responses (Xu and Zhang 2015). Though all endophytes found so far were classified as *Bacilli* members (phenotype/morphology; cultivation-independent *gyrA* sequence analyses), one cannot exclude the presence of other bacteria and possible effects on host features. MiSeq NGS analyses of metagenomics DNA from different cultivars should also clarify this question.

### Endophyte community and host: a functional unit?

Quinoa’s exceptional germination velocity and salt stress tolerance is related to a number of biochemical peculiarities. Being rich in protein and fat quinoa seeds provide an energy-rich environment to the developing embryo. Proteins behaving as ampholytes can assist water uptake during imbibition and thereby speed up the rehydration process. Catalase-mediated H_2_O_2_ detoxification ensures sufficient oxygenation during early development. A general limiting step in plant seedling development could be availability/mobilization of MAPK enzyme activities. Unlike other species, quinoa seeds deposit *active* forms of MAPKs. They are thus available for target substrate phosphorylation immediately upon rehydration. Noteworthy, MAPK substrate phosphorylation can drive cell expansion (Sasabe and Machida 2012). Cell wall-loosening functions of quinoa endophytes, including superoxide accumulation (Pitzschke 2016) cellulolytic and pectinolytic activities (Fig. 6a,b) suggest an active contribution of seed-borne bacteria to host cell expansion and - thus -rapid germination. Given the known role of ROS and cell wall fragments as potent, universal inducers of MAPK-mediated resistance (Boller and Felix 2009; Heil and Land 2014), quinoa endophytes have diverse tools to stimulate the host immunity system and thereby to put plants into a naturally primed state characterised by elevated stress resistance. Because of the obvious inexistence of non-inhabited seeds, plant functions cannot be separated from microbial functions. Much like “constitutively colonized” humans (Hutter et al. 2015) quinoa should better be considered as a super-individual or holobiont. Very likely, “optimal” bacterial assemblages preparing a host to environmental challenges vary, depending on stress type and habitat. Empirical optimization (qualitative and quantitative) of microbial inoculants for a given species and its cultivation condition seems a highly rewarding task. Compared to bacterial monocultures, such complex formulations appear superior for application in agriculture (Oslizlo et al. 2015), especially in climatically challenging conditions. Noteworthy in this context, terroir does not only drive microbial assemblages (Klaedtke et al. 2016) but – in turn - beneficial effects conferred by bacterial endophytes are also more evident in plants cultivated on marginal soils (Hardoim et al. 2012; Truyens et al. 2016a; Weyens et al. 2009). Cactus seeds whose endophytes had been found indispensable for seedling establishment on solid rock (Puente et al. 2009) would be an equally promising source of robust, potentially transferable beneficial inoculants.

### Bacterial factors mediating MAPK activation and host colonization

Plant growth-promoting rhizobacteria (PGPR) elicitors represent a diverse class of molecules that can mimic the perception of a pathogen and thus trigger elaborate defense responses in their host (Beneduzi et al. 2012). PeBA1, one such elicitor recently discovered in *Bacillus amyloliquefaciens* NC6, induces systemic resistance in tobacco by triggering early defense responses (Wang et al. 2016). Its application to tobacco leaves triggers ROS generation and accumulation of potentially barrier-forming phenolic compounds (Wang et al. 2016). PeBa1 could play a similar protective role in quinoa, given the phylogenetic relatedness of quinoa endophytes to *B. amyloliquefaciens* NC6 and presence of a PeBa1 homolog (Fig. 4c). Due to unavailability of non-colonized seed material, functionality of quinoa endophyte-derived elicitors such as flagellin, PeBA1 and BmyD will have to be demonstrated using purified peptides and non-native host organisms. Peptide purification experiments potentially also reveal further biofilm compounds such as surfactins, known as key players in *Bacillus* biofilm formation (Vlamakis et al. 2013). Interestingly, *Bacilli* strains co-inhabiting plant materials sense each other, communicate and work in concert for biofilm production (Oslizlo et al. 2015).

When cultivated on solid medium, quinoa-derived bacteria showed swarming and biofilm formation. Swarming is a multicellular movement powered by rotating helical flagella (Kearns 2010). Multicellular communication and multicellular growth might also be a strategy pursued by quinoa endophytes *in planta* that contributes to shaping the holobiont. Both flagelling and biofilm compounds potentially exert a double function, i.e. bacterial spreading and induction of host defense response. Biofilm components, lipopeptides from *Bacillus*, can trigger ISR in bean (Ongena et al. 2007), though it is still unclear whether this is directly linked to MAPK elicitation. Being capable of endospore formation and long-term survival in soil, biofilm-forming bacteria from quinoa possess key tools to colonize roots of other plant species. Noteworthy, maize root exudates have been found to stimulate biofilm production in *B. amyloliquefaciens* SQR9 (Zhang et al. 2015), the original source strain of bacillomycin D (Xu et al. 2013).

Quinoa endophytes are able of CMC- and pectin degradation (Fig. 6); functions associated with DAMP-mediated MAPK induction. These enzymatic properties would facilitate bacterial movement across cell walls – to migrate within quinoa, but also to enter new hosts after successful root colonization. Notably, despite cell wall-cleaving activities quinoa endophytes caused no plant tissue collapse or other obvious damage. Even high concentrations of (exogenously added) bacteria had no adverse effect on chia germination or seedling development (Fig. 6b). Bacterial culturing on CMC medium furthermore revealed that quinoa-derived bacteria can exploit cellulose derivatives as the only carbon and energy source. An inorganic form of nitrogen, here applied as NH_4_NO_3_, is sufficient. *B. amyloliquefaciens* strain SS35 isolated from animal dung behaves similar in that respect (Singh et al. 2013). Given that quinoa’s inhabitants are easy to cultivate and strong accumulators of superoxide, another promising research area involves exploring their potential for biofuel production.

## Conclusions

The more we know about the biochemistry and molecular mechanisms accompanying stress adaptation in crops, the better the chances for maintaining or improving agricultural production even under highly challenging conditions. This work on an exceptional halophyte crop provides a first and general image on quinoa MAPKs, and in particular on their activities during germination, a critical period in quinoa development (Adolf et al. 2013). The microbial partners have various chemical (Pitzschke 2016) and enzymatic (pectinase, cellulose) means to support host cell expansion driving embryonic axis growth and thus germination. Possible general growth-promoting effects will have to be tested by inoculation of non-native host species.

The results suggest a positive impact of quinoa endophytes on plant development and stress performance, which could partly be attributable to induction of host MAPKs. Considering their long-term survival, cell wall-penetrating functions, cell motility and the strong taxonomic relatedness to various beneficial (growth-promoting, ISR-inducing, fungal antagonists) quinoa endophytes hold high promise for improving productivity in transfected crop plants. Biofilm formation would assist colonization, though successful plant colonization and interaction depends on many more (reviewed in (Vacheron et al. 2013)). Noteworthy, quinoa endophytes can colonize chia seeds surfaces in large numbers without impairing germination or development. Whether colonization is sustainable and how it would affect MAPK-related signalling and stress performance in new host species still needs to be explored.

## Electronic supplementary material

ESM 1 (PDF 2241 kb)
