# Supplementary material for: Molecular dynamics in germinating, endophyte-colonized quinoa seeds
Source: Plant Soil. 2017 Feb 15;422(1):135–54. doi: 10.1007/s11104-017-3184-2 (PMC5798591; doi:10.1007/s11104-017-3184-2)
Supplement: Supplementary file 1 — (PDF 2241 kb) [file 11104_2017_3184_MOESM1_ESM.pdf]

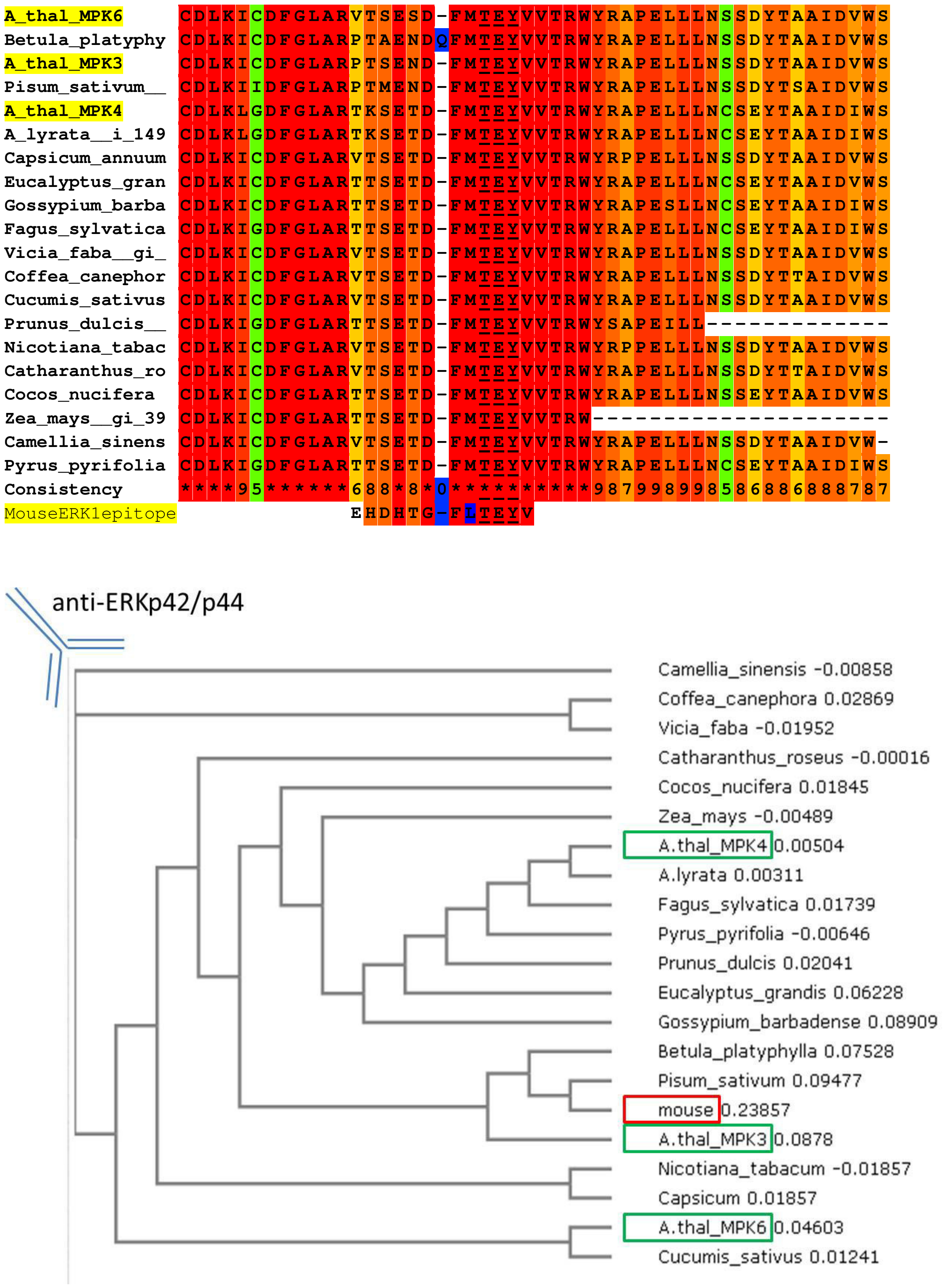

Suppl. Figure S1

Protein sequence alignment and phylogenetic tree of **MAPKs from diverse species** (PRALINE alignment tool; EMBL phylogeny tool). For reasons of clarity, the **dual phosphorylation motif (TEY)** plus highly conserved flanking regions are displayed. The peptide of mouse MAPK ERK1, presenting the **epitope for antibody** ERKp42/p44, is included in the alignment (bottom line). The antibody is reportedly suitable to detect active forms of *Arabidopsis* MPK3, MPK4 and MPK6 (highlighted).

Suppl. figure S2

Amino acid sequence alignment of the 18 putative quinoa MAPKs with all 20 Arabidopsis MAPKs.

The quinoa genome database (http://quinoa.kazusa.or.jp/) was screened for genes annotated as MAPKs, and the corresponding translation products were retrieved. Highly (red; 80%) and moderately (blue; 40%) conserved residues are shown in colour. The alignment was generated using the MultAlin tool. See also phylogenetic tree in figure 3.

Results colour-coded for amino acid conservation

The current colourscheme of the alignment is for **amino acid conservation**.

The conservation scoring is performed by PRALINE. The scoring scheme works from 0 for the least conserved alignment position, up to 10 for the most conserved alignment position. The colour assignments are:

Unconserved 0 1 2 3 4 5 6 7 8 9 10 **Conserved**

```
                 .........10 .........20 .........30 .........40 .........50
ATMPK11__B       ---------- ---------- ---------- ---------- ----------
ATMPK13__B       ---------- ---------- ---------- ---------- ----------
ATMPK6__A        ---------- ---------- ---------- ---------- ----------
ATMPK10__A       ---------- ---------- ---------- ---------- ----------
Cqu_c04092_1_g0  ------MGEA SPPGAYATVA AS-------W LGATT-TAWP SRLGAS---H
Cqu_c06022_1_g0  MVVADATAES VAALQYVLSH AMQDNDDLIL MHVTQPTKKS HLLIKSFFRR
Cqu_c03819_1_g0  ---------- ---------- ---------- ---------- ----------
Cqu_c04159_1_g0  ---------- ---------- ---------- ---------- ----------
ATMPK3__A        ---------- ---------- ---------- ---------- ----------
Cqu_c03402_1_g0  ---------- ---------- ---------- ---------- ----------
Cqu_c03736_1_g0  ---------- ---------- ---------- ---------- ----------
ATMPK12__B       ---------- ---------- ---------- ---------- ----------
ATMPK4__B        ---------- ---------- ---------- ---------- ----------
ATMPK5__B        ---------- ---------- ---------- ---------- ----------
Cqu_c17078_1_g0  ---------- ---------- ---------- ---------- ----------
ATMPK1__C        ---------- ---------- ---------- ---------- ----------
Cqu_c02858_1_g0  ---------- ---------- ---------- ---------- ----------
ATMPK7__C        ---------- ---------- ---------- ---------- ----------
ATMPK2__C        ---------- ---------- ---------- ---------- ----------
ATMPK14__C       ---------- ---------- ---------- ---------- ----------
ATMPK8__D        ---------- ---------- ---------- MGG------G GNLVDGVRRW
Cqu_c08055_1_g0  ---------- ---------- ---------- MGN------G -TLVDGVRR-
ATMPK15__D       ---------- ---------- ---------- MGG------G GNLVDGVRRW
ATMPK9__D        ---------- ---------- ---------- ---------- ----------
ATMPK18__D       ---------- ---------- ---------- ---------- ----------
ATMPK20__D       ---------- ---------- ---------- ---------- ----------
ATMPK19__D       ---------- ---------- ---------- ---------- ----------
Cqu_c06169_1_g0  ---------- ---------- ---------- ---------- ----------
Cqu_c03735_1_g0  ---------- ---------- ---------- ---------- ----------
ATMPK16__D       ---------- ---------- ---------- ---------- ----------
Cqu_c07086_1_g0  ---------- ---------- ---------- ---------- ----------
Cqu_c09059_1_g0  ---------- ---------- ---------- ---------- ----------
ATMPK17__D       ---------- ---------- ---------- ---------- ----------
Cqu_c04094_1_g0  ---------- ---------- ---------- ---------- ----------
Cqu_c01012_1_g0  ---------- ---------- ---------- MG-------N GTLVDGVRRW
Cqu_c14619_1_g0  ---------- ---------- ---------- ---------- ----------
Cqu_c03126_1_g0  ---------- ---------- ---------- ---------- ----------
Cqu_c01012_1_g0  ---------- ---------- ---------- ---------- ----------
Consistency      0000000000 0000000000 0000000000 0000000000 0000000000

                 .........60 .........70 .........80 .........90 .........100
ATMPK11__B       ---------- ---------- ---------- ---------- ----------
ATMPK13__B       ---------- ---------- ---------- ---------- ----------
ATMPK6__A        ---------- ---------- ---------- ---------- ----------
ATMPK10__A       ---------- ---------- ---------- ---------- ----------
Cqu_c04092_1_g0  H--------- -FVLT----- ---------- IRYEPR---- -FP------A
Cqu_c06022_1_g0  HSDPGLAGIS NFDPTADVGE GGGGSGGKDF LEVMKRACKG TFPNSRVWVE
Cqu_c03819_1_g0  ---------- ---------- ---------- ---------- ----------
Cqu_c04159_1_g0  ---------- ---------- ---------- ---------- ----------
ATMPK3__A        ---------- ---------- ---------- ---------- ----------
Cqu_c03402_1_g0  ---------- ---------- ---------- ---------- ----------
Cqu_c03736_1_g0  ---------- ---------- ---------- ---------- ----------
ATMPK12__B       ---------- ---------- ---------- ---------- ----------
ATMPK4__B        ---------- ---------- ---------- ---------- ----------
ATMPK5__B        ---------- ---------- ---------- ---------- ----------
Cqu_c17078_1_g0  ---------- ---------- ---------- ---------- ----------
ATMPK1__C        ---------- ---------- ---------- ---------- ----------
Cqu_c02858_1_g0  ---------- ---------- ---------- ---------- ----------
ATMPK7__C        ---------- ---------- ---------- ---------- ----------
ATMPK2__C        ---------- ---------- ---------- ---------- ----------
ATMPK14__C       ---------- ---------- ---------- ---------- ----------
ATMPK8__D        -L-------- -FQRPS---- -------SSS SSSSSNNNNN NHEQPIFNSS
Cqu_c08055_1_g0  -W-------- -FSRRG---- -------GVV ANNSYKLDES INNNNSSNSS
ATMPK15__D       LF-------- -FQRRP---- -------SSS SSSNNHDQIQ NP--------
```

```
ATMPK9__D        ---------- ---------- ---------- ---------- ----------
ATMPK18__D       ---------- ---------- ---------- ---------- ----------
ATMPK20__D       ---------- ---------- ---------- ---------- ----------
ATMPK19__D       ---------- ---------- ---------- ---------- ----------
Cqu_c06169_1_g0  ---------- ---------- ---------- ---------- ----------
Cqu_c03735_1_g0  ---------- ---------- ---------- ---------- ----------
ATMPK16__D       ---------- ---------- ---------- ---------- ----------
Cqu_c07086_1_g0  ---------- ---------- ---------- ---------- ----------
Cqu_c09059_1_g0  ---------- ---------- ---------- ---------- ----------
ATMPK17__D       ---------- ---------- ---------- ---------- ----------
Cqu_c04094_1_g0  ---------- ---------- ---------- ---------- ----------
Cqu_c01012_1_g0  ---------- -FSRRG---- -------VVV ANNSYKLDES INNNNSSNSS
Cqu_c14619_1_g0  ---------- ---------- ---------- ---------- ----------
Cqu_c03126_1_g0  ---------- ---------- ---------- ---------- ----------
Cqu_c01012_1_g0  ---------- ---------- ---------- ---------- ----------
Consistency      0000000000 0000000000 0000000000 0000000000 0000000000

                 .........110.......120.........130.........140.........150
ATMPK11__B       ---------- ---------- ---------- ---------- ----------
ATMPK13__B       ---------- ---------- ---------- ---------- ----------
ATMPK6__A        ---------- ---------- ---------- -------MDG G-SGQPAADT
ATMPK10__A       ---------- ---------- ---------- -------ME- -----PTNDA
Cqu_c04092_1_g0  TMA------- ----TLRCIS PPTTSLRVSE SSDTHTIMDA S--AAQPSDT
Cqu_c06022_1_g0  KVEQGSLDNK GAVILLRCKE LMVDLLVVGQ RRHLVSVLLG PRRSFSTGST
Cqu_c03819_1_g0  ---------- ---------- ---------- ---------- ----------
Cqu_c04159_1_g0  ---------- ---------- ---------- ---------- ----------
ATMPK3__A        ---------- ---------- ---------- ---------- ----------
Cqu_c03402_1_g0  ---------- ---------- ---------- ---------- ----------
Cqu_c03736_1_g0  ---------- ---------- ---------- ---------- ----------
ATMPK12__B       ---------- ---------- ---------- ---------- ----------
ATMPK4__B        ---------- ---------- ---------- ---------- ----------
ATMPK5__B        ---------- ---------- ---------- ---------- ----------
Cqu_c17078_1_g0  ---------- ---------- ---------- ---------- ----------
ATMPK1__C        ---------- ---------- ---------- ---------- ----------
Cqu_c02858_1_g0  ---------- ---------- ---------- ---------- ----------
ATMPK7__C        ---------- ---------- ---------- ---------- ----------
ATMPK2__C        ---------- ---------- ---------- ---------- ----------
ATMPK14__C       ---------- ---------- ---------- ---------- ----------
ATMPK8__D        SF-------- -------SSS SNPNHSANSG ELIIEEDLDF S-------GL
Cqu_c08055_1_g0  SSRQVSVISE FSGNQINNHS SSKQQEGEEQ ELNIEFDFDL S-------GL
ATMPK15__D       ---------- ---------- --PTVSNPND DEDLKKLTDP S-------KL
ATMPK9__D        ---------- ---------- ---------- ---------- ----------
ATMPK18__D       ---------- ---------- ---------- ---------- ----------
ATMPK20__D       ---------- ---------- ---------- ---------- ----------
ATMPK19__D       ---------- ---------- ---------- ---------- ----------
Cqu_c06169_1_g0  ---------- ---------- ---------- ---------- ----------
Cqu_c03735_1_g0  ---------- ---------- ---------- ---------- ----------
ATMPK16__D       ---------- ---------- ---------- ---------- ----------
Cqu_c07086_1_g0  ---------- ---------- ---------- ---------- ----------
Cqu_c09059_1_g0  ---------- ---------- ---------- ---------- ----------
ATMPK17__D       ---------- ---------- ---------- ---------- ----------
Cqu_c04094_1_g0  ---------- ---------- -MDASAAQPS DTDMMDAAGA PPENHPPPSS
Cqu_c01012_1_g0  SSR------- --QVSVISEF SGKQINNHSS SKQQGEEEEQ Q-EFNIEFDF
Cqu_c14619_1_g0  ---------- ---------- ---------- ---------- ----------
Cqu_c03126_1_g0  ---------- ---------- ---------- ---------- ----------
Cqu_c01012_1_g0  ---------- ---------- ---------- ---------- ----------
Consistency      0000000000 0000000000 0000000000 0000000000 0000000000

                 .........160.........170.........180.........190.........200
ATMPK11__B       -MSIEKPF-- ---------- ---------- -FGDDSNRGV SINGGRYVQY
ATMPK13__B       ---------- ---------- ---------- -MEKREDGGI LTYDGRYVMY
ATMPK6__A        EMTEAPGGFP --AAA----- ------PSPQ MPGIENIPAT LSHGGRFIQY
ATMPK10__A       ETLETQGEVT TAIWP----- ------SSQI LKTTIDIPGT LSHDGRYIQY
Cqu_c04092_1_g0  DMMDAAGAPP ENHPP-PSSD QSQPPQQPPQ QQQQVSIPAT LSHGGKFIQY
Cqu_c06022_1_g0  VLRGGAPKSV DMVEYLIENS PCTCVAVQKK GQNSGYILNS KTHRNFWLLY
Cqu_c03819_1_g0  -MSEFPMAAA ---------- -------AGG GGQFPEFPAV YTHGGQFIQY
Cqu_c04159_1_g0  -MSEFPMAAA ---------- -------AGG GGQFPEFPAV YTHGGQFIQY
ATMPK3__A        ---------- ---------- ------MNTG GGQYTDFPAV ETHGGQFISY
Cqu_c03402_1_g0  ---------- ---------- ------ME-N SAKSKEVRGI PTYGGTYTMY
Cqu_c03736_1_g0  ---------- ---------- ---------- --MEQNLRGV PTHGGRYVQY
ATMPK12__B       -MSGESSS-- ---------- ---------- GSTEHCIKVV PTHGGRYVQY
ATMPK4__B        -MSAESCFG- ---------- ---------S SGDQSSSKGV ATHGGSYVQY
ATMPK5__B        -MAKEIESA- ---------- ---------T DLGDTNIKGV LVHGGRYFQY
Cqu_c17078_1_g0  ---------- ---------- -------MEN SAKSKEVRGI PTYGGTYILY
ATMPK1__C        ---------- ---------- ---------- MATLVDPPNG IRNEGK-HYF
```

```
Cqu_c02858_1_g0 ---------- ---------- ---------- MATQVEPPNG VRCPGK-HYY
ATMPK7__C       ---------- ---------- ---------- MAMLVEPPNG IKQQGK-HYY
ATMPK2__C       ---------- ---------- ---------- MATPVDPPNG IRNQGK-HYF
ATMPK14__C      ---------- ---------- ---------- MAMLVDPPNG IRQEGK-HYY
ATMPK8__D       TLINVPKR-- ---------- ---------- ----NHLPMD PHK----KGE
Cqu_c08055_1_g0 KPIKVPKR-- ---------- ---------- -APTSRFPLP MDPHKKSALE
ATMPK15__D      RQIKVQQR-- ---------- ---------- ----NHLPME KKG----IPN
ATMPK9__D       ---------- ---------- ---------- ------MDPH KKV----ALE
ATMPK18__D      ------M--- ---------- ---------- -----QQNQV KKG----TKE
ATMPK20__D      ------M--- ---------- ---------- -----QQDNR KKN----NLE
ATMPK19__D      ------M--- ---------- ---------- -----QKTQE KKN----MKE
Cqu_c06169_1_g0 ------M--- ---------- ---------- -----QQDQR KKA----VKE
Cqu_c03735_1_g0 MEQNLRG--- ---------- ---------- -----TPTHG GRY----VQY
ATMPK16__D      ------M--- ---------- ---------- -----QPDHR KKS----SVE
Cqu_c07086_1_g0 ------M--- ---------- ---------- -----QQDQR KKT----SVD
Cqu_c09059_1_g0 ---------- ---------- ---------- ----MQQDQR KKA----VKE
ATMPK17__D      ---------- ---------- ---------- ---------- -------MLE
Cqu_c04094_1_g0 DQSQPPQQP- ---------- --------PQ QQQQVSIPAT LSHGGKFIQY
Cqu_c01012_1_g0 DLSGLKPIK- ---------- -------VPK RAPTSRFPLP MDPHKKSALE
Cqu_c14619_1_g0 ---------- ---------- ---------- ---------- ----------
Cqu_c03126_1_g0 ---------- ---------- ---------- ---------- ----------
Cqu_c01012_1_g0 ---------- ---------- ---------- ---------- ----------
Consistency     0000001000 0000000000 0000000000 1111132332 3431221434

                .........210.........220.........230.........240.........250
ATMPK11__B      NVYGNLFEVS KKYVPPLRPI GRGASGIVCA AWNSETGEEV AIKKIGNAFG
ATMPK13__B      NVLGNIFELS SKYIPPIEPI GRGAYGIVCC ATNSETNEEV AIKKIANAFD
ATMPK6__A       NIFGNIFEVT AKYKPPIMPI GKGAYGIVCS AMNSETNESV AIKKIANAFD
ATMPK10__A      NLFGHIFELP AKYKPPIRPI GRGACGIVCS AVDSETNEKV AIKKITQVFD
Cqu_c04092_1_g0 NIFGNIFEVT AKYKPPIMPI GKGAYGIVCS SLNSDTNEHV AIKKIANAFD
Cqu_c06022_1_g0 NVHGSLFEVS SKYVPPIRPI GRGASGLVCA AVNSDTHHEV AIKKIGNAFD
Cqu_c03819_1_g0 NIFGNLFEVT AKYRPPIMPI GRGAYGIVCS VLNTETKEMV ALKKIANAFD
Cqu_c04159_1_g0 NIFGNLFEVT AKYRPPIMPI GRGAYGIVCS VLNTETKEMV ALKKIANAFD
ATMPK3__A       DIFGSLFEIT SKYRPPIIPI GRGAYGIVCS VLDTETNELV AMKKIANAFD
Cqu_c03402_1_g0 NILGTLFEVS SKYAPPIQPV GRGAYGIVCC ARNSETNEDV AIKKIADAFN
Cqu_c03736_1_g0 NLYGNLFEVS SKYVPPLRPV GRGAYGIVCA AVNSETQEEV AIKKVSNAFH
ATMPK12__B      NVYGQLFEVS RKYVPPIRPI GRGACGIVCA AVNSVTGEKV AIKKIGNAFD
ATMPK4__B       NVYGNLFEVS RKYVPPLRPI GRGAYGIVCA ATNSETGEEV AIKKIGNAFD
ATMPK5__B       NVYGNLFEVS NKYVPPIRPI GRGAYGFVCA AVDSETHEEI AIKKIGKAFD
Cqu_c17078_1_g0 NIFGSLFEVA SKYAPPIQPV GRGAYGIVCC ARNSETNEDV AIKKIADAFN
ATMPK1__C       SMWQTLFEID TKYM-PIKPI GRGAYGVVCS SVNSDTNEKV AIKKIHNVYE
Cqu_c02858_1_g0 SMWQALFEID TKYV-PIKPI GRGAYGIVCS SXNKETNERV AIKKIHNAFE
ATMPK7__C       SMWQTLFEID TKYV-PIKPI GRGAYGVVCS SINRETNERV AIKKIHNVFE
ATMPK2__C       SMWQTLFEID TKYV-PIKPI GRGAYGVVCS SVNRESNERV AIKKIHNVFE
ATMPK14__C      TMWQTLFEID TKYV-PIKPI GRGAYGVVCS SINSETNERV AIKKIHNVFE
ATMPK8__D       TEFFTEYGEA NRYQ-IQEVV GKGSYGVVAS AVDSHTGERV AIKKINDVFE
Cqu_c08055_1_g0 NDFFTEYGEA SRYQ-VQEII GKGSYGIVGS AVDTHTGEXV AIKKINDVFE
ATMPK15__D      AEFFTEYGEA NRYQ-IQEVV GKGSYGVVGS AIDTHTGERV AIKKINDVFD
ATMPK9__D       TEFFTEYGEA SRYQ-IQEVI GKGSYGVVAS AIDTHSGEKV AIKKINDVFE
ATMPK18__D      MEFFTEYGDA NRYR-ILEVI GKGSYGVVCA AIDTHTGEKV AIKKINDVFE
ATMPK20__D      MEFFSDYGDA NRFK-VQEVI GKGSYGVVCS AIDTLTGEKV AIKKIHDIFE
ATMPK19__D      MEFFTEYGDA NRYR-ILEVI GKGSYGVVCA AIDTQTGEKV AIKKINDVFE
Cqu_c06169_1_g0 PEFFTDYGEA NRYK-ILEVI GKGSYGVVCA AIDTHTGEKV AIKKINDIYE
Cqu_c03735_1_g0 NLYGNVFEVS SKYVPPLRPV GRGAYGIVCA AVNSETQEEV AIKKVSNAFH
ATMPK16__D      VDFFTEYGEG SRYR-IEEVI GKGSYGVVCS AYDTHTGEKV AIKKINDIFE
Cqu_c07086_1_g0 VDFFTEYGEG NRYK-IEEVI GKGSYGVVCS AYDTHVGEKV AXKKINDIFE
Cqu_c09059_1_g0 PEFFTDYGEA NRYK-ILEVI GKGSYGVVCA AIDTHTGEKV AIKXINDXYE
ATMPK17__D      KEFFTEYGEA SQYQ-IQEVV GKGSYGVVAS AECPHTGGKV AIKKMTNVFE
Cqu_c04094_1_g0 NIFGNIFEVT AKYKPPIMPI GKGAYGIVCS SLNSDTNEHV AIKKIANAFD
Cqu_c01012_1_g0 NDFFTEYGEA SRYQ-VQEII GKGSYGIVGS AVDTHTGEKV AIKKINDVFE
Cqu_c14619_1_g0 ---------- ---------- ---------- ---------- ----------
Cqu_c03126_1_g0 ---------- ---------- ---------- ---------- ----------
Cqu_c01012_1_g0 ---------- ---------- ---------- ---------- -----MDWYP
Consistency     4453547544 5684145458 8786687866 6555474748 8788846686

                .........260.........270.........280.........290.........300
ATMPK11__B      NIIDAKRTLR EIKLLKHMDH -DNVIAI-ID IIRPPQPDNF NDVHIVYELM
ATMPK13__B      NRVDAKRTLR EIKLLSHMDH -DNVIKI-KD IIELPEKERF EDVYIVYELM
ATMPK6__A       NKIDAKRTLR EIKLLRHMDH -ENIVAI-RD IIPPPLRNAF NDVYIAYELM
ATMPK10__A      NTIEAKRTLR EIKLLRHFDH -ENIVAI-RD VILPPQRDSF EDVYIVNELM
Cqu_c04092_1_g0 NKVDAKRTLR EIKLLRHMDH -ENVVAI-RD IIPPPRREAF NDVYXAYELM
Cqu_c06022_1_g0 NIVDAKRTLR EIKLLRHMDH -ENIIAI-KD IIRPPKKEAF NDVYIVYELM
Cqu_c03819_1_g0 NYMDAKRTLR EIKILRHFDH -ENIIVL-RD VIPPPVRREF TDVYVATELM
Cqu_c04159_1_g0 NYMDAKRTLR EIKILRHFDH -ENIIVL-RD VIPPPVRREF TDVYVATELM
ATMPK3__A       NHMDAKRTLR EIKLLRHLDH -ENIIAI-RD VVPPPLRRQF SDVYISTELM
```

```
Cqu_c03402_1_g0 NRIDAKRTLR EIKLLCHMDH -DNVIKI-KD IIRPPERENF NDVYIVYELL
Cqu_c03736_1_g0 NRIDAKRILR EIKLLRHMNH -DNVISI-RD IIRPPQKENF NDVYMVHELV
ATMPK12__B      NIIDAKRTLR EIKLLRHMDH -ENVITI-KD IVRPPQRDIF NDVYIVYELM
ATMPK4__B       NIIDAKRTLR EIKLLKHMDH -ENVIAV-KD IIKPPQRENF NDVYIVYELM
ATMPK5__B       NKVDAKRTLR EIKLLRHLEH -ENVVVI-KD IIRPPKKEDF VDVYIVFELM
Cqu_c17078_1_g0 NRIDAKRTLR EIKLLCHMDH -DNVIKI-KD IIRPPERENF NDVYIVYELL
ATMPK1__C       NRIDALRTLR ELKLLRHLRH -ENVIAL-KD VMMPIHKMSF KDVYLVYELM
Cqu_c02858_1_g0 NRVDALRTLR ELKLLRHLRH -DNVIAL-KD VMLPAHPKNF KDVYLVYELM
ATMPK7__C       NRVDALRTLR ELKLLRHVRH -ENVIAL-KD VMLPANRSSF KDVYLVYELM
ATMPK2__C       NRIDALRTLR ELKLLRHLRH -ENVVAL-KD VMMANHKRSF KDVYLVYELM
ATMPK14__C      NRIDALRTLR ELKLLRHVRH -ENVISL-KD VMLPTHRYSF RDVYLVYELM
ATMPK8__D       HVSDATRILR EIKLLRLLRH -PDVVEI-KH IMLPPSRREF RDIYVVFELM
Cqu_c08055_1_g0 HVSDATRILR EIKLLRLLRH -PDVVEI-KH IMLPPSRREF KDIYVVFELM
ATMPK15__D      HISDATRILR EIKLLRLLLH -PDVVEI-KH IMLPPSRREF RDVYVVFELM
ATMPK9__D       HVSDATRILR EIKLLRLLRH -PDIVEI-KH VMLPPSRREF RDIYVVFELM
ATMPK18__D      HISDALRILR EVKLLRLLRH -PDIVEI-KS IMLPPSKREF KDIYVVFELM
ATMPK20__D      HISDAARILR EIKLLRLLRH -PDIVEI-KH IMLPPSRREF KDIYVVFELM
ATMPK19__D      HVSDALRILR EVKLLRLLRH -PDIVEI-KS IMLPPSKREF KDIYVVFELM
Cqu_c06169_1_g0 HISDAIRILR EVKLLRLLRH -PDIVEI-KR IMXPPSKRDF RDIYVVFELM
Cqu_c03735_1_g0 NRIDAKRILR EIKLLRHMNH -DNVISI-RD IIRPPQKENF NDVYMVHELM
ATMPK16__D      HVSDATRILR EIKLLRLLRH -PDIVEI-KH ILLPPSRREF RDIYVVFELM
Cqu_c07086_1_g0 HVSDATRILR EIKLLRLLRH -PDIVEI-KH ILLPPSRREF KDIYVVFELM
Cqu_c09059_1_g0 HISDAIXILR EVKLLRLLRH -PDIVEI-KR IMLPPSKRDF RDIYVVLELM
ATMPK17__D      HVSDAIRILR EIKLLRLLRH -PDIVEI-KH IMLPPCRKEF KDIYVVFELM
Cqu_c04094_1_g0 NKVDAKRTLR EIKLLRHMDH -ENVVAI-RD IIPPPRREAF NDVYIAYELM
Cqu_c01012_1_g0 HVSDATRILR EIKLLRLLHF -AHET----- ---------- ----------
Cqu_c14619_1_g0 ---------- ---------- ---------- ---------- ----------
Cqu_c03126_1_g0 ---------- ---------- ---------- ---------- ----------
Cqu_c01012_1_g0 GHGVDRFKRQ FAHLEEHLGK GEKSTPLSRQ HASLPRERVP APKTDANENN
Consistency     6458848588 8788875648 0567747065 7637636447 4877665887

                .........310.......320.......330.......340.......350
ATMPK11__B      DTDLHHIIRS NQPLTDDHSR FFLYQLLRGL KYVHSANVLH RDLKPSNLLL
ATMPK13__B      DTDLHQIIRS TQTLTDDHCQ YFLYQILRGL KYIHSANVLH RDLKPSNLVL
ATMPK6__A       DTDLHQIIRS NQALSEEHCQ YFLYQILRGL KYIHSANVLH RDLKPSNLLL
ATMPK10__A      EFDLYRTLKS DQELTKDHGM YFMYQILRGL KYIHSANVLH RDLKPSNLLL
Cqu_c04092_1_g0 DTDLHQIIRS NQGLSEEHCQ YFLYQXLRGL KYIHSANVLH RDLKPSNLLL
Cqu_c06022_1_g0 DTDLHQIIRS SQALTDDHCQ YFLYQLLRGL KYVHSAKVLH RDLKPSNLLL
Cqu_c03819_1_g0 DTDLHQIIRS HQSLSEEHCQ YFLYQILRGL KYIHSANVIH RDLKPSNLLI
Cqu_c04159_1_g0 DTDLHQIIRS HQSLSEEHCQ YFLYQILRGL KYIHSANVIH RDLKPSNLLI
ATMPK3__A       DTDLHQIIRS NQSLSEEHCQ YFLYQLLRGL KYIHSANIIH RDLKPSNLLL
Cqu_c03402_1_g0 DTDLHQIIRS TQELTEDHCQ YFLYQILRGL KYIHSANVLH RDLKPSNLLL
Cqu_c03736_1_g0 DTDLHQIIRS NQQLTDDHCR YFLYQILRGL KYIHSAGVLH RDLKPSNLLM
ATMPK12__B      DTDLQRILRS NQTLTSDQCR FLVYQLLRGL KYVHSANILH RDLRPSNVLL
ATMPK4__B       DTDLHQIIRS NQPLTDDHCR FFLYQLLRGL KYVHSANVLH RDLKPSNLLL
ATMPK5__B       DTDLHQIIRS NQSLNDDHCQ YFLYQILRGL KYIHSANVLH RDLKPSNLLL
Cqu_c17078_1_g0 DTDLHQIIRS TQELTEDHCQ YFLYQILRGL KYIHSANVLH RDLKPSNLLL
ATMPK1__C       DTDLHQIIKS SQVLSNDHCQ YFLFQLLRGL KYIHSANILH RDLKPGNLLV
Cqu_c02858_1_g0 DTDLHQIXKS SQALSNDHCQ YFLFQLLRGL KYLHSANILH RDLKPGNLLI
ATMPK7__C       DTDLHQIIKS SQSLSDDHCK YFLFQLLRGL KYLHSANILH RDLKPGNLLV
ATMPK2__C       DTDLHQIIKS SQVLSNDHCQ YFLFQLLRGL KYIHSANILH RDLKPGNLLV
ATMPK14__C      DSDLNQIIKS SQSLSDDHCK YFLFQLLRGL KYLHSANILH RDLKPGNLLV
ATMPK8__D       ESDLHQVIKA NDDLTPEHYQ FFLYQLLRGL KYVHAANVFH RDLKPKNILA
Cqu_c08055_1_g0 ESDLHQVIKA NDDXTPEHYQ FFLYQLLRGL KYVHTANVFH RDLKPKNILA
ATMPK15__D      ESDLHQVIKA NDDLTPEHHQ FFLYQLLRGL KYVHAANVFH RDLKPKNILA
ATMPK9__D       ESDLHQVIKA NDDLTPEHYQ FFLYQLLRGL KFIHTANVFH RDLKPKNILA
ATMPK18__D      ESDLHQVIKA NDDLTREHHQ FFLYQMLRAL KFMHTANVYH RDLKPKNILA
ATMPK20__D      ESDLHQVIKA NDDLTREHYQ FFLYQLLRAL KYIHTANVYH RDLKPKNILA
ATMPK19__D      ESDLHQVIKA NDDLTREHHQ FFLYQMLRAL KYMHTANVYH RDLKPKNILA
Cqu_c06169_1_g0 ESDLHQVIKA NDDLTREHHQ FFLYQMLRAL KYMHTANVYH RDLKPKNILA
Cqu_c03735_1_g0 DTDLHQIIRS NQQLTDDHCR YFLYQILRGL KYIHSAGVLH RDLKPSNLLM
ATMPK16__D      ESDLHQVIKA NDDLTPEHYQ FFLYQLLRGL KYIHTANVFH RDLKPKNILA
Cqu_c07086_1_g0 ESDXHQVIKA NDDLTPEHYQ FFLYQLLRGL KYIHTANVFH RDLKPKNILA
Cqu_c09059_1_g0 ESDLHQVIKA NDDLTREHHQ FFLYQMLRAL KYMHTANVYH RDLKPKNILA
ATMPK17__D      ESDLHHVLKV NDDLTPQHHQ FFLYQLLRGL KFMHSAHVFH RDLKPKNILA
Cqu_c04094_1_g0 DTDLHQIIRS NQGLSEEHCQ YFLYQILRGL KYNIFCQCSY RDLKPSNLCL
Cqu_c01012_1_g0 ---------- ------RPCS VALTTDDQIF SSSYAANVFH RDLKPKNILA
Cqu_c14619_1_g0 ---------- ---------- ---------- ---------- ----------
Cqu_c03126_1_g0 ---------- ---------- ---------- ---------- ----------
Cqu_c01012_1_g0 DMENRAVART ALE-SPPKSN ADGSENEVPQ NGQNKGNYSA RSLLKSASIS
Consistency     6687777776 5547646746 6787868868 8768687758 8888858785

                .........360.......370.......380.......390.......400
ATMPK11__B      NANCDLKIGD FGLARTK--- -SETDFMTEY VVTRWYRAPE LLLNC-SEYT
ATMPK13__B      NTNCDLKICD FGLARTS--- -NETEIMTEY VVTRWYRAPE LLLNS-SEYT
```

```
ATMPK6__A       NANCDLKICD FGLARVT--- -SESDFMTEY VVTRWYRAPE LLLNS-SDYT
ATMPK10__A      STQCDLKICD FGLARAT--- -PESNLMTEY VVTRWYRAPE LLLGS-SDYT
Cqu_c04092_1_g0 NANCDLKICD FGLARVT--- -SETDFMTEY VVTRWYRAPE LLLNS-SDYT
Cqu_c06022_1_g0 NANCDLKIGD FGLARTT--- -TETDFMTEY VVTRWYRAPE LLLNC-SEYT
Cqu_c03819_1_g0 NANCDLKICD FGLARPT--- -SENEHMTEY VVTRWYRAPE LLLNS-SDYT
Cqu_c04159_1_g0 NANCDLKICD FGLARPT--- -SENEHMTEY VVTRWYRAPE LLLNS-SDYT
ATMPK3__A       NANCDLKICD FGLARPT--- -SENDFMTEY VVTRWYRAPE LLLNS-SDYT
Cqu_c03402_1_g0 NANCDLKICD FGLARTT--- -AETDFMTEY VVTRWYRAPE LLLNC-SEYT
Cqu_c03736_1_g0 NANCDLKIGD FGLARTT--- -SETDFMTEY VVTRWYRAPE LLLNC-SEYT
ATMPK12__B      NSKNELKIGD FGLARTT--- -SDTDFMTEY VVTRWYRAPE LLLNC-SEYT
ATMPK4__B       NANCDLKLGD FGLARTK--- -SETDFMTEY VVTRWYRAPE LLLNC-SEYT
ATMPK5__B       NSNCDLKITD FGLARTT--- -SETEYMTEY VVTRWYRAPE LLLNS-SEYT
Cqu_c17078_1_g0 NANCDLKICD FGLARTT--- -AETDFMTEY VVTRWYRAPE LLLNC-SEYT
ATMPK1__C       NANCDLKICD FGLARAS--- NTKGQFMTEY VVTRWYRAPE LLLCC-DNYG
Cqu_c02858_1_g0 NANCDLKICD FGLARTS--- SGKGQFMTEY VVTRWYRAPE LLLCC-DKYG
ATMPK7__C       NANCDLKICD FGLARTS--- QGNEQFMTEY VVTRWYRAPE LLLCC-DNYG
ATMPK2__C       NANCDLKICD FGLARTS--- NTKGQFMTEY VVTRWYRAPE LLLCC-DNYG
ATMPK14__C      NANCDLKICD FGLART---- --YEQFMTEY VVTRWYRAPE LLLCC-DNYG
ATMPK8__D       NADCKLKICD FGLARVSFND APTAIFWTDY VATRWYRAPE LCGSFFSKYT
Cqu_c08055_1_g0 NADCKLKICD FGLARVSFND APSAIFWTDY VATRWYRAPE LCGSFFSKYT
ATMPK15__D      NADCKLKICD FGLARVSFND APTAIFWTDY VATRWYRAPE LCGSFFSKYT
ATMPK9__D       NSDCKLKICD FGLARVSFND APSAIFWTDY VATRWYRAPE LCGSFFSKYT
ATMPK18__D      NANCKLKVCD FGLARVAFND TPTTVFWTDY VATRWYRAPE LCGSFFSKYT
ATMPK20__D      NANCKLKICD FGLARVAFND TPTTIFWTDY VATRWYRAPE LCGSFYSKYT
ATMPK19__D      NANCKLKVCD FGLARVSFND TPTTVFWTDY VATRWYRAPE LCGSFCSKYT
Cqu_c06169_1_g0 NANCKLKICD FGLARVAFND TPTTIFWTDY VATRWYRAPE LCGSFFSKYT
Cqu_c03735_1_g0 NANCDLKIGD FGLART---- TSETDFMTEY VVTRWYRAPE LLLN-CSEYT
ATMPK16__D      NADCKLKICD FGLARVAFND TPTAIFWTDY VATRWYRAPE LCGSFFSKYT
Cqu_c07086_1_g0 NADCKLKICD FGLARVAFND TPTAIFWTDY VATRWYRAPE LCGSFFSKYT
Cqu_c09059_1_g0 NANCKLKICD FGLARVAFND TPTTIFWTDY VATRWYRAPE LCGSFFSKYT
ATMPK17__D      NADCKIKICD LGLARVSFTD SPSAVFWTDY VATRWYRAPE LCGSFYSNYT
Cqu_c04094_1_g0 ---------- ---------- ---------- MPTRWYRAPE LLLN------
Cqu_c01012_1_g0 NADCKLKICD FGLARVSFND APSAIFWTDY VATRWYRAPE LCGSFFSKYT
Cqu_c14619_1_g0 ---------- ---------- ---------- ---------- --------YT
Cqu_c03126_1_g0 ---------- -MLYFHT--- TTGESFLTDY VVMRWYKEPE L---------
Cqu_c01012_1_g0 GSKC-IGV-- ---------- ---------- -QSRKDPEEE PIAE------
Consistency     7768588857 7888855111 1444375868 8689998899 9545306486

                .........410.........420.........430.........440.........450
ATMPK11__B      AAIDIWSVGC ILGEIMT--- --REPLFPGR DYVQQLRLIT ELIGSPDDSS
ATMPK13__B      GAIDIWSVGC IFMEILR--- --RETLFPGK DYVQQLKLIT ELLGSPDDSD
ATMPK6__A       AAIDVWSVGC IFMELMD--- --RKPLFPGR DHVHQLRLLM ELIGTPSEEE
ATMPK10__A      AAIDVWSVGC IFMEIMN--- --REPLFPGK DQVNQLRLLL ELIGTPSEEE
Cqu_c04092_1_g0 AAIDVWSVGC IFMELMD--- --RKPLFPGR DHVHQLRLLM ELIGTPSEHE
Cqu_c06022_1_g0 AAIDVWSVGC IFGEIMT--- --REPLFPGK DYVHQLRLIT ELIGSPDDSS
Cqu_c03819_1_g0 ASIDLWSVGC IFMELMN--- --RRPLFPGK DHVHQMRLLT ELLGTPTEAD
Cqu_c04159_1_g0 ASIDLWSVGC IFMELMN--- --RKPLFPGR DHVHQMRLLT ELLGTPTEAD
ATMPK3__A       AAIDVWSVGC IFMELMN--- --RKPLFPGK DHVHQMRLLT ELLGTPTESD
Cqu_c03402_1_g0 AAIDMWSVGC IMMEIIK--- --REPLFPGK DYLQQLQLIN ELLGSPEDSD
Cqu_c03736_1_g0 AAIDTWSVGC ILGEIMT--- --REPLFPGK DYVHQLKLVT ELIGSPDDSS
ATMPK12__B      AAIDIWSVGC ILGEIMT--- --GQPLFPGK DYVHQLRLIT ELVGSPDNSS
ATMPK4__B       AAIDIWSVGC ILGETMT--- --REPLFPGK DYVHQLRLIT ELIGSPDDSS
ATMPK5__B       SAIDVWSVGC IFAEIMT--- --REPLFPGK DYVHQLKLIT ELIGSPDGAS
Cqu_c17078_1_g0 AAIDMWSVGC IMMEIIK--- --KEPLFPGK DYLQQLQLIN ELLGSPEDSD
ATMPK1__C       TSIDVWSVGC IFAELLG--- --RKPIFQGT ECLNQLKLIV NILGSQREED
Cqu_c02858_1_g0 TSIDVWSVGC IFAELLG--- --RKPLFPGT ECLNQLKLIV NILGSQRDAD
ATMPK7__C       TSIDVWSVGC IFAEILG--- --RKPIFPGT ECLNQLKLII NVVGSQQESD
ATMPK2__C       TSIDVWSVGC IFAELLG--- --RKPVFPGT ECLNQIKLII NILGSQREED
ATMPK14__C      TSIDVWSVGC IFAEILG--- --RKPIFPGT ECLNQLKLII NVVGSQQDWD
ATMPK8__D       PAIDIWSVGC IFAEMLL--- --GKPLFPGK NVVHQLDLMT DFLGTPPPES
Cqu_c08055_1_g0 PAIDIWSIGC IFAEMLT--- --GKPLFPGK NVVHQLDLMT DLMGTPPPEA
ATMPK15__D      PAIDIWSVGC IFAEMLL--- --GKPLFPGK NVVHQLDIMT DFLGTPPPEA
ATMPK9__D       PAIDIWSIGC IFAEMLT--- --GKPLFPGK NVVHQLDIMT DLLGTPPPEA
ATMPK18__D      PAIDVWSIGC IFAEVLT--- --GKPLFPGK SVVHQLELIT DLLGTPKSET
ATMPK20__D      PAIDIWSIGC IFAEVLM--- --GKPLFPGK NVVHQLDLMT DLLGTPSLDT
ATMPK19__D      PAIDIWSIGC IFAEVLT--- --GKPLFPGK SVVHQLDLIT DLLGTPKSET
Cqu_c06169_1_g0 PAIDIWSIGC IFAEVLT--- --GKPLFPGK SVVHQLDIIT DLLGTPSHDV
Cqu_c03735_1_g0 AAIDIWSVGC ILGEIMT--- --REPLFPGR DYVHQLKLVT ELIGSPDDSS
ATMPK16__D      PAIDIWSIGC IFAELLT--- --GKPLFPGK NVVHQLDLMT DMLGTPSAEA
Cqu_c07086_1_g0 PAIDIWSIGC IFAELLT--- --GKPLFPGK NVVHQLDLMT ELLGTPSPDA
Cqu_c09059_1_g0 PAIDIWSIGC IFAEVLT--- --GKPLFPGK SVVHQLDIIT DLLGTPSHDV
ATMPK17__D      PAIDMWSVGC IFAEMLT--- --GKPLFPGK NVVHQLELVT DLLGTPSPIT
Cqu_c04094_1_g0 -SSDLQLLSM YVEDALA--- --HHYLNSLH DISDEPICMS -------PFC
Cqu_c01012_1_g0 PAIDIWSIGV YLQKCSLENH YSWKKCGASV GPMDR----- -SYGDSTP--
Cqu_c14619_1_g0 PAIDIWSIGC IFAELLT--- --GKPLFPGK NVVHQLDLMT ELLGTPSPDA
```

```
Cqu_c03126_1_g0 ------QIKF IINFL----- ---------- ---------- ---GSPSESD
Cqu_c01012_1_g0 NEEAVDELTK KATALAT--- ---------- ---------- ----------
Consistency     4789789988 8758774000 0046788786 5376874765 5668674444

                .........460.........470.........480.........490.........500
ATMPK11__B      LGFLRSDNAR RYVRQLPQYP RQNFAARFPN MSVNAVDLLQ KMLVFDPNRR
ATMPK13__B      LDFLRSDNAR KYVKQLPHVQ KQSFREKFPN ISPMALDLAE KMLVFDPSKR
ATMPK6__A       LEFL-NENAK RYIRQLPPYP RQSITDKFPT VHPLAIDLIE KMLTFDPRRR
ATMPK10__A      LGSL-SEYAK RYIRQLPTLP RQSFTEKFPN VPPLAIDLVE KMLTFDPKQR
Cqu_c04092_1_g0 LGFL-NENAK RYIRQLPPFR RQSLSEKFPX VHPAAIDLIE KMLTFDPRQR
Cqu_c06022_1_g0 LGFLRSNHAR RYVKQLPQYP KQQFSARFPN MSPAATDLLE KMLVFDPNKR
Cqu_c03819_1_g0 LGFLHNEDAR RYIRQLPPQP RQQLRQVFPH VNPLAIDLIE KMLTFDPTRR
Cqu_c04159_1_g0 LGFLHNEDAR RYIRQLPPQP RQQLRQLFPH VNPLAIDLIE RMLTFDPTRR
ATMPK3__A       LGFTHNEDAK RYIRQLPNFP RQPLAKLFSH VNPMAIDLVD RMLTFDPNRR
Cqu_c03402_1_g0 LGFLRSENAK KYIKQLPHVP KQPFSHKFPS VSPLVLDLAE KMLVFDPSKR
Cqu_c03736_1_g0 IAFLQSENAK RYVRQLPQYP RQQFSVKFPN ASPLAIDLLE KMLVFDPSKR
ATMPK12__B      LGFLRSDNAR RYVRQLPRYP KQQFAARFPK MPTTAIDLLE RMLVFDPNRR
ATMPK4__B       LGFLRSDNAR RYVRQLPQYP RQNFAARFPN MSAGAVDLLE KMLVFDPSRR
ATMPK5__B       LEFLRSANAR KYVKELPKFP RQNFSARFPS MNSTAIDLLE KMLVFDPVKR
Cqu_c17078_1_g0 LGFLRSENAK KYIKQLPHVP KQPFSHKFPS VSPLVLDLAE KMLVFDPSKR
ATMPK1__C       LEFIDNPKAK RYIRSLPYSP GMSLSRLYPG AHVLAIDLLQ KMLVFDPSKR
Cqu_c02858_1_g0 LEFIDNPKAK RFIKSLPYSP TASLSRLYPT ANPLAIDLLQ KMLVFDPTKR
ATMPK7__C       IRFIDNPKAR RFIKSLPYSR GTHLSNLYPQ ANPLAIDLLQ RMLVFDPTKR
ATMPK2__C       LEFIDNPKAK RYIESLPYSP GISFSRLYPG ANVLAIDLLQ KMLVLDPSKR
ATMPK14__C      LQFIDNQKAR RFIKSLPFSK GTHFSHIYPH ANPLAIDLLQ RMLVFDPTKR
ATMPK8__D       ISRIRNEKAR RYLSSMRKKQ PVPFSHKFPK ADPLALRLLE RLLAFDPKDR
Cqu_c08055_1_g0 ISRIRNEKAR RYLSXMRKKP PIPFSQKFPN VDPLALRLLE RLLAFDPKDR
ATMPK15__D      ISKIRNDKAR RYLGNMRKKQ PVPFSKKFPK ADPSALRLLE RLIAFDPKDR
ATMPK9__D       IARIRNEKAR RYLGNMRRKP PVPFTHKFPH VDPLALRLLH RLLAFDPKDR
ATMPK18__D      ISGVRNDKAR KYLTEMRKKN PVTFSQKFSK ADPLALRLLQ RLLAFDPKDR
ATMPK20__D      ISRVRNEKAR RYLTSMRKKP PIPFAQKFPN ADPLSLKLLE RLLAFDPKDR
ATMPK19__D      IAGVRNEKAR KYLNEMRKKN LVPFSQKFPN ADPLALRLLQ RLLAFDPKDR
Cqu_c06169_1_g0 ISGVRNDKAR KYLTDMRKKQ PVPFSHKFSN ADPLALRLLQ RLLSFDPKDR
Cqu_c03735_1_g0 IAFLQSENAK RYVRQLPQYP RQQFSVKFPN ASPLAIDLLE KMLVFDPSKR
ATMPK16__D      IGRVRNEKAR RYLSSMRKKK PIPFSHKFPH TDPLALRLLE KMLSFEPKDR
Cqu_c07086_1_g0 IARIRNEXAR RYLSSMRKKK PIPFTHKFPN ADPLALRLLE RMLAFEPKDR
Cqu_c09059_1_g0 ISGVRNDKAR KYLTDMRKKQ PVPFSHKFSN ADPLALRLLQ RLLSFDPKDR
ATMPK17__D      LSRIRNEKAR KYLGNMRRKD PVPFTHKFPN IDPVALKLLQ RLIAFDPKDR
Cqu_c04094_1_g0 FDFEQHALSE EQMRELIYRE ALAFNPEFQQ E--------- ----------
Cqu_c01012_1_g0 ---------- ---------- ---------- ---------- ----------
Cqu_c14619_1_g0 IAPG-LSRSS PIISIMRKKK AYSFHHKFPN ADPLAFVCWN EMLAVEPKDR
Cqu_c03126_1_g0 LAFIKNQNVV KFIEGMPYSN GVRLEDEYLW ANPLALDLLR RMLVLNPTKR
Cqu_c01012_1_g0 ---------- ---------- ---------- ---------- ----------
Consistency     7446465586 7774575344 3447535874 5465765866 6785778448

                .........510.........520.........530.........540.........550
ATMPK11__B      ITVDEALCHP YLAPLHEYNE --------EPV C--VRPFHFD F-EQPSLTEE
ATMPK13__B      ITVDEALKQP YLASLHEINE --------EPT C--PTPFSFD F-EETALDEQ
ATMPK6__A       ITVLDALAHP YLNSLHDISD --------EPE C--TIPFNFD F-ENHALSEE
ATMPK10__A      ISVKEALAHP YLSSFHDITD --------EPE C--SEPFNFD L-DEHPFSEE
Cqu_c04092_1_g0 ITVEDALAHH YLNSLHDISD --------EPI C--MSPFCFD F-EQHALSEE
Cqu_c06022_1_g0 VTVDEALCHP YLASLHDIND --------EPI C--SAPFRFD F-EQSSLTEE
Cqu_c03819_1_g0 ITVEEALAHP YLARLHDIAD --------EPV C--QEPFSFD F-ENQALTEE
Cqu_c04159_1_g0 ITVEEALAHP YLARLHDIAD --------EPV C--QEPFSFD F-ENQALTEE
ATMPK3__A       ITVEQALNHQ YLAKLHDPND --------EPI C--QKPFSFE F-EQQPLDEE
Cqu_c03402_1_g0 ITVDEALNHP YLSSLHEINE --------EPT C--PKPFQFD F-EQSTLDEE
Cqu_c03736_1_g0 ITVHEALCHP YLAPLHDINE --------EPV C--PQPFSFD F-EQPSFTEE
ATMPK12__B      ISVDEALGHA YLSPHHDVAK --------EPV C--STPFSFD F-EHPSCTEE
ATMPK4__B       ITVDEALCHP YLAPLHDINE --------EPV C--VRPFNFD F-EQPTLTEE
ATMPK5__B       ITVEEALCYP YLSALHDLND --------EPV C--SNHFSFH F-EDPSSTEE
Cqu_c17078_1_g0 ITVDEALNHP YLSSLHEINE --------EPT C--PKPFQFD F-EQSTLDEE
ATMPK1__C       ISVSEALQHP YMAPLYDPNA --------NPP A--QVPIDLD V-DED-LREE
Cqu_c02858_1_g0 ISVTEALQHP YMSPLYDPRA --------NPP A--QFPIDLE I-DED-LXEE
ATMPK7__C       ISVTDALLHP YMAGLFDPGS --------NPP A--HVPISLD I-DEN-MEEP
ATMPK2__C       ISVTEALQHP YMAPLYDPSA --------NPP A--QVPIDLD VDEDEDLGAE
ATMPK14__C      ISVSDALLHP YMEGLLEPEC --------NPS E--NVPVSSL E-IDENMEGD
ATMPK8__D       ASAEDALADP YFSGLSNSER --------EPT TQPISKLEFD F-ERKKLVKD
Cqu_c08055_1_g0 PSAAEALTDP YFYGLSSADR --------EPC TTPISKLEFE F-ERRKLAKD
ATMPK15__D      PSAEEALADP YFNGLSSKVR --------EPS TQPISKLEFE F-ERKKLTKD
ATMPK9__D       PSAEEALADP YFYGLANVDR --------EPS TQPIPKLEFE F-ERRKITKE
ATMPK18__D      PTPAEALADP YFKGLSKIER --------EPS SQQISKMEFE F-ERRRLTKD
ATMPK20__D      PTAEEALADP YFKGLAKVER --------EPS CQPITKMEFE F-ERRKVTKE
ATMPK19__D      PTAAEALADP YFKCLAKVER --------EPS CQPISKMEFE F-ERRRLTKD
Cqu_c06169_1_g0 PTAEEALADP YFKGLSRVER --------EPA CQPISKLEFE F-ERRRVTKE
Cqu_c03735_1_g0 ITVHEALCHP YLAPLHDINE --------EPV C--PQPFSFD F-EQPSFTEE
```

```
ATMPK16__D      PTAEEALADV YFKGLAKVER -------EPS AQPVTKLEFE F-ERRRITKE
Cqu_c07086_1_g0 PSAEEALADP YFKGLAKVER -------EPS AQPVTKMEFE F-ERRRMTKE
Cqu_c09059_1_g0 PTAEELL-KD YMNGNEGSNY ------IYPS A--IGQFKKQ F-AHLEENAG
ATMPK17__D      PSAEEALADP YFQGLANVDY -------EPS RQPISKLEFE F-ERRKLTRD
Cqu_c04094_1_g0 ---------- ---------- ---------- ---------- ----------
Cqu_c01012_1_g0 ---------- ---------- ---------- ---------- ----------
Cqu_c14619_1_g0 PSAEEALADP YFKGLAKFAY LEEHYKNGST GPPIDRQHAS L-PRACVLYS
Cqu_c03126_1_g0 ITVTEALRHS YMADLHIPTE -------YPP P--QRSLDID I--EADVGES
Cqu_c01012_1_g0 ---------- ---------- ---------- ---------- ----------
Consistency     4664788356 8543744443 0000000683 4003355466 6064335456

                .........560.........570.........580.........590.........600
ATMPK11__B      NIKELIYRES VKFNP----- ---------- ---------- ----------
ATMPK13__B      DIKELVWRES LHFKNM---- ---------- ---------- ----------
ATMPK6__A       QMKELIYREA LAFNPEYQQ- ---------- ---------- ----------
ATMPK10__A      QFRELIYCEA LAFNPETSND ---------- ---------- ----------
Cqu_c04092_1_g0 QMRELIYREA LAFNPEFQQE ---------- ---------- ----------
Cqu_c06022_1_g0 NVKELIWKES VKFNPDPAH- ---------- ---------- ----------
Cqu_c03819_1_g0 QMKDMIHQEA LALNPGYI-- ---------- ---------- ----------
Cqu_c04159_1_g0 QMKDMIHQEA LALNPGYI-- ---------- ---------- ----------
ATMPK3__A       QIKEMIYQEA IALNPTYG-- ---------- ---------- ----------
Cqu_c03402_1_g0 DIKELVWRET LKFNPDKMIE ---------- ---------- ----------
Cqu_c03736_1_g0 NIKELIWRES VSFNPDPLIG ---------- ---------- ----------
ATMPK12__B      HIKELIYKES VKFNPDH--- ---------- ---------- ----------
ATMPK4__B       NIKELIYRET VKFNPQDSV- ---------- ---------- ----------
ATMPK5__B       EIKELVWLES VKFNPLPSI- ---------- ---------- ----------
Cqu_c17078_1_g0 DIKELVWRET LKFNPDKMIE ---------- ---------- ----------
ATMPK1__C       MIREMMWNEM LHYHPQASTL -NT----EL- ---------- ----------
Cqu_c02858_1_g0 MIRESMWQEI MHYHPEAINQ -NM----ELC G--------- ----------
ATMPK7__C       VIREMMWNEM LYYHPEAEIS -NA------- ---------- ----------
ATMPK2__C       MIRELMWKEM IHYHPEAATI NNN----EVS EF-------- ----------
ATMPK14__C      MIREMMWEEM LHYLPRA--- ---------- ---------- ----------
ATMPK8__D       DVRELIYREI LEYHPQMLEE YLRG-G-DQL SFMYPSGVDR FK----RQFA
Cqu_c08055_1_g0 DVRELIYREI LEYHPQMLQE YLRG-GQDQT SFMYPSGVDR FK----RQFA
ATMPK15__D      DIRELIYREI LEYHPQMLEE YLRG-G-NQL SFMYPSGVDR FR----RQFA
ATMPK9__D       DVRELIYREI LEYHPQMLQE YLRG-G-EQT SFMYPSGVDR FK----RQFA
ATMPK18__D      DIRELIYREI LEYHPQLLKD YMSG--SEGS NFVYPSAIGH LR----QQFT
ATMPK20__D      DIRELISREI LEYHPQLLKD HMNG--ADKA SFLYPSAVDQ FR----RQFA
ATMPK19__D      DIRELIYREI LEYHPQLLKD YMNS---EGS SFLYPSAIGH LR----KQFA
Cqu_c06169_1_g0 DVRELIYREI LEYHPQLLKD YMNG--NEGS NYIYPSAIGQ FK----KQFA
Cqu_c03735_1_g0 NIKELIWRES VSEQHQFLMH FLDRHPLESL PVAAP-RLPH FKTLCTSEIQ
ATMPK16__D      DVRELIYRES LEYHPKMLKE YLDG--SEPT NFMYPSAVEH FK----KQFA
Cqu_c07086_1_g0 DVRELIYREI LEYHPKMLKE FLDG--SEPT NFMYPSAVDH FK----KQFA
Cqu_c09059_1_g0 RSGPVIPLER KH--ISLPRS TVH----SST IPPKPLASSH ------KDHQ
ATMPK17__D      DVRELMYREI LEYHPQMLQE YLQGEENINS HFLYPSGVDQ FK----QEFA
Cqu_c04094_1_g0 ---------- ---------- ---------- ---------- ----------
Cqu_c01012_1_g0 ---------- ---------- ---------- ---------- ----------
Cqu_c14619_1_g0 DNSVHGSTEV TEDLSKCRIK EV-------- ---------- ------EKAH
Cqu_c03126_1_g0 YLRELIWREM VF-------- ---------- ---------- ----------
Cqu_c01012_1_g0 ---------- ---------- ---------- ---------- ----------
Consistency     4667665584 6454642321 0100000100 0001000000 0000000100

                .........610.........620.........630.........640.........650
ATMPK11__B      ---------- ---------- ---------- ---------- ----------
ATMPK13__B      ---------- ---------- ---------- ---------- ----------
ATMPK6__A       ---------- ---------- ---------- ---------- ----------
ATMPK10__A      ---------- ---------- ---------- ---------- ----------
Cqu_c04092_1_g0 ---------- ---------- ---------- ---------- ----------
Cqu_c06022_1_g0 ---------- ---------- ---------- ---------- ----------
Cqu_c03819_1_g0 ---------- ---------- ---------- ---------- ----------
Cqu_c04159_1_g0 ---------- ---------- ---------- ---------- ----------
ATMPK3__A       ---------- ---------- ---------- ---------- ----------
Cqu_c03402_1_g0 ---------- ---------- ---------- ---------- ----------
Cqu_c03736_1_g0 ---------- ---------- ---------- ---------- ----------
ATMPK12__B      ---------- ---------- ---------- ---------- ----------
ATMPK4__B       ---------- ---------- ---------- ---------- ----------
ATMPK5__B       ---------- ---------- ---------- ---------- ----------
Cqu_c17078_1_g0 ---------- ---------- ---------- ---------- ----------
ATMPK1__C       ---------- ---------- ---------- ---------- ----------
Cqu_c02858_1_g0 ---------- ---------- ---------- ---------- ----------
ATMPK7__C       ---------- ---------- ---------- ---------- ----------
ATMPK2__C       ---------- ---------- ---------- ---------- ----------
ATMPK14__C      ---------- ---------- ---------- ---------- ----------
ATMPK8__D       HLEENQGKPG AAGGGRSTA- ---LHRHHAS LPRERVPAPN G---------
Cqu_c08055_1_g0 HLEEHLGK-- ---GEKSTP- ---LSRQHAS LPRERVPAPK T---------
```

```
ATMPK15__D        HLEENQGP-- ---GGRSNA- ---LQRQHAS LPRERVPASK N---------
ATMPK9__D         HLEENYGK-- ---GEKGSP- ---LQRQHAS LPRERVPAPK K---------
ATMPK18__D        YLEENSSRN- ---G-PVIP- ---LERKHAS LPRSTVHSTV VHSTS-----
ATMPK20__D        HLEENSGKT- ---G-PVAP- ---LERKHAS LPRSTVIHST AVARG-----
ATMPK19__D        YLEENSGKS- ---G-PVIP- ---PDRKHAS LPRSAVHSSA VNSNA-----
Cqu_c06169_1_g0   HLEENAGRS- ---G-PVIP- ---LERKHIS LPRSTVHSST IPPKP-----
Cqu_c03735_1_g0   IMKNSYGLV- ---Y-PSIPS RAGPKRRNSF MRGITCTSSS NSAFFLPNIP
ATMPK16__D        YLEEHYKNG- ---TSHNPP- ---ERQQHAS LPRACVLYSD NNHPV-----
Cqu_c07086_1_g0   YLEEHYKNG- ---S-TGPP- ---IDRQHAS LPRACVLYSD NSV-------
Cqu_c09059_1_g0   AKEE------ ---------- ---------- -MRPSESYG- ----------
ATMPK17__D        RLEEHNDDEE ---EHNSPP- ---HQRKYTS LPRERVCSSE ----------
Cqu_c04094_1_g0   ---------- ---------- ---------- ---------- ----------
Cqu_c01012_1_g0   ---------- ---------- ---------- ---------- ----------
Cqu_c14619_1_g0   VDRNG----- ------AIP- ---MTRLPLQ VPQN------ ----------
Cqu_c03126_1_g0   ---------- ---------- ---------- ---------- ----------
Cqu_c01012_1_g0   ---------- ---------- ---------- ---------- ----------
Consistency       0011000000 0000000000 0000010000 0110000000 0000000000

                  .........660.........670.........680.........690.........700
ATMPK11__B        ---------- ---------- ---------- ---------- ----------
ATMPK13__B        ---------- ---------- ---------- ---------- ----------
ATMPK6__A         ---------- ---------- ---------- ---------- ----------
ATMPK10__A        ---------- ---------- ---------- ---------- ----------
Cqu_c04092_1_g0   ---------- ---------- ---------- ---------- ----------
Cqu_c06022_1_g0   ---------- ---------- ---------- ---------- ----------
Cqu_c03819_1_g0   ---------- ---------- ---------- ---------- ----------
Cqu_c04159_1_g0   ---------- ---------- ---------- ---------- ----------
ATMPK3__A         ---------- ---------- ---------- ---------- ----------
Cqu_c03402_1_g0   ---------- ---------- ---------- ---------- ----------
Cqu_c03736_1_g0   ---------- ---------- ---------- ---------- ----------
ATMPK12__B        ---------- ---------- ---------- ---------- ----------
ATMPK4__B         ---------- ---------- ---------- ---------- ----------
ATMPK5__B         ---------- ---------- ---------- ---------- ----------
Cqu_c17078_1_g0   ---------- ---------- ---------- ---------- ----------
ATMPK1__C         ---------- ---------- ---------- ---------- ----------
Cqu_c02858_1_g0   ---------- ---------- ---------- ---------- ----------
ATMPK7__C         ---------- ---------- ---------- ---------- ----------
ATMPK2__C         ---------- ---------- ---------- ---------- ----------
ATMPK14__C        ---------- ---------- ---------- ---------- ----------
ATMPK8__D         -ETAEESSDV ERRAAAAVAS T--------- ---------- ---------L
Cqu_c08055_1_g0   -DANENNDME NRAVARTALE S--------- ---------- ---------P
ATMPK15__D        -ETVEERSND IERRTTAAVA S--------- ---------- ---------T
ATMPK9__D         -ENGSHNHDI ENRSIASLVT T--------- ---------- ---------L
ATMPK18__D        -QPNLGATDS RRVSFEPSKN GASSAGHPST SAYPTKSIGP PPRVPP---S
ATMPK20__D        -GQPKLMNNT NTLNPETTQN IP----FNHA TIQA-----Q QRNLS----A
ATMPK19__D        -QPSLNASDS RRVSIEPSRN GV----VPST SAYSTKPLGP PPRVP----S
Cqu_c06169_1_g0   -LASSHKDHQ AKEEMRPSES YG----NLSS SIR------Q PPRVPP---A
Cqu_c03735_1_g0   GIIALLEDLN LLINGDPQMI PSLGKHIPVQ EAGNQTKVFG YYRFPPLNQR
ATMPK16__D        -AQQSSAEVT DGLSKCSIRD ERPRGADRNA QMPMSRIPIN VPQTIQGAAV
Cqu_c07086_1_g0   ---HGSTEVT EDLSKCRIKE VEKAHVDRNG AIPMTRLPLQ VPQNVQ-GAA
Cqu_c09059_1_g0   -NLSSSIRQP PRVPP----- ---------- ---------- ---------A
ATMPK17__D        -DEGSDSVHA QSSSASVVFT P--------- ---------- ---------P
Cqu_c04094_1_g0   ---------- ---------- ---------- ---------- ----------
Cqu_c01012_1_g0   ---------- ---------- ---------- ---------- ----------
Cqu_c14619_1_g0   -VQGAAARPG RF-------- ---------- ---------- ----------
Cqu_c03126_1_g0   ---------- ---------- ---------- ---------- ----------
Cqu_c01012_1_g0   ---------- ---------- ---------- ---------- ----------
Consistency       0000000000 0000000000 0000000000 0000000000 0000000000

                  .........710.........720.........730.........740.........750
ATMPK11__B        ---------- ---------- ---------- ---------- ----------
ATMPK13__B        ---------- ---------- ---------- ---------- ----------
ATMPK6__A         ---------- ---------- ---------- ---------- ----------
ATMPK10__A        ---------- ---------- ---------- ---------- ----------
Cqu_c04092_1_g0   ---------- ---------- ---------- ---------- ----------
Cqu_c06022_1_g0   ---------- ---------- ---------- ---------- ----------
Cqu_c03819_1_g0   ---------- ---------- ---------- ---------- ----------
Cqu_c04159_1_g0   ---------- ---------- ---------- ---------- ----------
ATMPK3__A         ---------- ---------- ---------- ---------- ----------
Cqu_c03402_1_g0   ---------- ---------- ---------- ---------- ----------
Cqu_c03736_1_g0   ---------- ---------- ---------- ---------- ----------
ATMPK12__B        ---------- ---------- ---------- ---------- ----------
ATMPK4__B         ---------- ---------- ---------- ---------- ----------
ATMPK5__B         ---------- ---------- ---------- ---------- ----------
Cqu_c17078_1_g0   ---------- ---------- ---------- ---------- ----------
```

```
ATMPK1__C        ---------- ---------- ---------- ---------- ----------
Cqu_c02858_1_g0  ---------- ---------- ---------- ---------- ----------
ATMPK7__C        ---------- ---------- ---------- ---------- ----------
ATMPK2__C        ---------- ---------- ---------- ---------- ----------
ATMPK14__C       ---------- ---------- ---------- ---------- ----------
ATMPK8__D        ESEE------ -AD------- ----NGGGYS ARNLMKSASI ---SGSKCIG
Cqu_c08055_1_g0  PKSN------ -ADGSENEVP QNGQNKGNYS ARSLLKSASI ---SGSKCIG
ATMPK15__D       LDSP------ -KASQQAE-G TENGGGGGYS ARNLMKSSSI ---SGSKCIG
ATMPK9__D        ESPP------ -TSQHEGSDY RNGTSQTGYS ARSLLKSASI ---SASKCIG
ATMPK18__D       GRPGRVV--- -ESSVSYENG RNLKEAYFRS -AVSSPHCYF RPNTMTNPEN
ATMPK20__D       AKPSTFM--- -GPVAPFDNG RISRDAYDP- -RSFIRSTNL PFSQQSAATV
ATMPK19__D       GKPGRVV--- -ESSVTYEND RNLKESSYDA RTSYYRSTVL PPQTVSPNCY
Cqu_c06169_1_g0  ARPGRVV--- -GPVVAYESG RSMKDGYDA- -RVSLQNAVI PPQTASPQYY
Cqu_c03735_1_g0  SDFEKYALCS PGLQYAFEAD KCLNKSFADS YHNFDFPTTH ANDNLRPIAN
ATMPK16__D       ARPGKVV--- -GSVLRYNNC -GAATGVEAL EQQQRRMVRN PAAASQYPKR
Cqu_c07086_1_g0  ARPGKVV--- -GNLMRYNNC -GVAATAEVT DRRPGRNPSI PASQFPTPSS
Cqu_c09059_1_g0  ARPGRVV--- -GPVAYESG- --RSMKDGYD ARVSLQNAVI PPQTASPQYY
ATMPK17__D       QTPNTAT--- -GLSSQKASQ VDKAATPVKR SACLMRSDSI ---CASRCVG
Cqu_c04094_1_g0  ---------- ---------- ---------- ---------- ----------
Cqu_c01012_1_g0  ---------- ---------- ---------- ---------- ----------
Cqu_c14619_1_g0  ---------- ---------- ---------- ---------- ---SETLCVI
Cqu_c03126_1_g0  ---------- ---------- ---------- ---------- ----------
Cqu_c01012_1_g0  ---------- ---------- ---------- ---------- ----------
Consistency      0000000000 0000000000 0000000000 0000000000 0000000000

                 .........760.........770.........780.........790.........800
ATMPK11__B       ---------- ---------- ---------- ---------- ----------
ATMPK13__B       ---------- ---------- ---------- ---------- ----------
ATMPK6__A        ---------- ---------- ---------- ---------- ----------
ATMPK10__A       ---------- ---------- ---------- ---------- ----------
Cqu_c04092_1_g0  ---------- ---------- ---------- ---------- ----------
Cqu_c06022_1_g0  ---------- ---------- ---------- ---------- ----------
Cqu_c03819_1_g0  ---------- ---------- ---------- ---------- ----------
Cqu_c04159_1_g0  ---------- ---------- ---------- ---------- ----------
ATMPK3__A        ---------- ---------- ---------- ---------- ----------
Cqu_c03402_1_g0  ---------- ---------- ---------- ---------- ----------
Cqu_c03736_1_g0  ---------- ---------- ---------- ---------- ----------
ATMPK12__B       ---------- ---------- ---------- ---------- ----------
ATMPK4__B        ---------- ---------- ---------- ---------- ----------
ATMPK5__B        ---------- ---------- ---------- ---------- ----------
Cqu_c17078_1_g0  ---------- ---------- ---------- ---------- ----------
ATMPK1__C        ---------- ---------- ---------- ---------- ----------
Cqu_c02858_1_g0  ---------- ---------- ---------- ---------- ----------
ATMPK7__C        ---------- ---------- ---------- ---------- ----------
ATMPK2__C        ---------- ---------- ---------- ---------- ----------
ATMPK14__C       ---------- ---------- ---------- ---------- ----------
ATMPK8__D        VQSKTDKEDT IAEEEDNETV AELTDKVASL HNS------- ----------
Cqu_c08055_1_g0  VQSRKDPEEE PI-AENEEAV DELTKKATAL AT-------- ----------
ATMPK15__D       VQSKTNIED- ---SIVEEQD ETVAVKVASL HNS------- ----------
ATMPK9__D        MKPRNKSEYG ---ESNNDTV DALSQKVAAL HT-------- ----------
ATMPK18__D       RNIEASSFPP KPQNPVHQ-- ---FSPTEPP AATTNQADVE TMNHPNPYFQ
ATMPK20__D       AMGKQQERRT ---TMEPE-- ---KQARQIS QYNRYAPDVA INIDNNPFIM
ATMPK19__D       FLPNTMNQEK RSGTEAAS-- ---QPKPQFV PTQ-CNSAKP AELNPNPYVQ
Cqu_c06169_1_g0  IRPNIQPAQS KPGLGAGNEQ GHVSWAKQQQ LPVQPTHEVT IDINTNPYHH
Cqu_c03735_1_g0  FLHMNNSMAW FHQVKAVAAT VAKYFTGPLL PRRSLLATQL IREPFFPLLS
ATMPK16__D       TQPCKSNRGD EDCATAAEGP S--RLKPN-T QYIPQKVSAA QDTAMSRWY-
Cqu_c07086_1_g0  SYPKRNPGCK NERGEDDSME GSNGLQPK-P QYLARKVAAA QAGPGSQWY-
Cqu_c09059_1_g0  IRPNIQPAQS KPGLGAGNEQ GHVSWAKQQQ LPVQPTHEVT IDINTNPYHH
ATMPK17__D       VSSAVS---- ---------- ---------- ---------- ----------
Cqu_c04094_1_g0  ---------- ---------- ---------- ---------- ----------
Cqu_c01012_1_g0  ---------- ---------- ---------- ---------- ----------
Cqu_c14619_1_g0  NNCGVAATAE VTDRRPGEIH RYLPLNSLHL PKPQYLARKV AAAQAGP---
Cqu_c03126_1_g0  ---------- ---------- ---------- ---------- ----------
Cqu_c01012_1_g0  ---------- ---------- ---------- ---------- ----------
Consistency      0000000000 0000000000 0000000000 0000000000 0000000000

                 .........810.........820.........830.........840.........850
ATMPK11__B       ---------- ---------- ---------- ---------- ----------
ATMPK13__B       ---------- ---------- ---------- ---------- ----------
ATMPK6__A        ---------- ---------- ---------- ---------- ----------
ATMPK10__A       ---------- ---------- ---------- ---------- ----------
Cqu_c04092_1_g0  ---------- ---------- ---------- ---------- ----------
Cqu_c06022_1_g0  ---------- ---------- ---------- ---------- ----------
Cqu_c03819_1_g0  ---------- ---------- ---------- ---------- ----------
Cqu_c04159_1_g0  ---------- ---------- ---------- ---------- ----------
```

```
ATMPK3__A         ---------- ---------- ---------- ---------- ----------
Cqu_c03402_1_g0   ---------- ---------- ---------- ---------- ----------
Cqu_c03736_1_g0   ---------- ---------- ---------- ---------- ----------
ATMPK12__B        ---------- ---------- ---------- ---------- ----------
ATMPK4__B         ---------- ---------- ---------- ---------- ----------
ATMPK5__B         ---------- ---------- ---------- ---------- ----------
Cqu_c17078_1_g0   ---------- ---------- ---------- ---------- ----------
ATMPK1__C         ---------- ---------- ---------- ---------- ----------
Cqu_c02858_1_g0   ---------- ---------- ---------- ---------- ----------
ATMPK7__C         ---------- ---------- ---------- ---------- ----------
ATMPK2__C         ---------- ---------- ---------- ---------- ----------
ATMPK14__C        ---------- ---------- ---------- ---------- ----------
ATMPK8__D         ---------- ---------- ---------- ---------- ----------
Cqu_c08055_1_g0   ---------- ---------- ---------- ---------- ----------
ATMPK15__D        ---------- ---------- ---------- ---------- ----------
ATMPK9__D         ---------- ---------- ---------- ---------- ----------
ATMPK18__D        PQLPKTDQLN NNTHMAIDAK LLQAQ----- --SQFG-PAG AA---AVAVA
ATMPK20__D        ARTGMNKAEN ISDRIIIDTN LLQAT----- --AGIG-VAA AAAAAAPGGS
ATMPK19__D        SQH------- ---KVGIDAK LLHAQ----- --SQYG-PAG AA---AVAVA
Cqu_c06169_1_g0   PQPKSDQ--- YSSRVALDAK IMQAQ----- -----S-QFS AT---AVAMS
Cqu_c03735_1_g0   ASPPVTNPNP HTEALAALAQ THIQMFIGHP YLSQFGHPVG SGPFWAFRPI
ATMPK16__D        ---------- ---------- ---------- ---------- ----------
Cqu_c07086_1_g0   ---------- ---------- ---------- ---------- ----------
Cqu_c09059_1_g0   PQPKSDQ--- YSSRVALDAK IMQAQS---- -------QFS AT---AVAMS
ATMPK17__D        ---------- ---------- ---------- ---------- ----------
Cqu_c04094_1_g0   ---------- ---------- ---------- ---------- ----------
Cqu_c01012_1_g0   ---------- ---------- ---------- ---------- ----------
Cqu_c14619_1_g0   ---------- --GSQGTKCT YLQFW----- ---------- ----------
Cqu_c03126_1_g0   ---------- ---------- ---------- ---------- ----------
Cqu_c01012_1_g0   ---------- ---------- ---------- ---------- ----------
Consistency       0000000000 0000000000 0000000000 0000000000 0000000000

                  .........860.........870.....
ATMPK11__B        ---------- ---------- -----
ATMPK13__B        ---------- ---------- -----
ATMPK6__A         ---------- ---------- -----
ATMPK10__A        ---------- ---------- -----
Cqu_c04092_1_g0   ---------- ---------- -----
Cqu_c06022_1_g0   ---------- ---------- -----
Cqu_c03819_1_g0   ---------- ---------- -----
Cqu_c04159_1_g0   ---------- ---------- -----
ATMPK3__A         ---------- ---------- -----
Cqu_c03402_1_g0   ---------- ---------- -----
Cqu_c03736_1_g0   ---------- ---------- -----
ATMPK12__B        ---------- ---------- -----
ATMPK4__B         ---------- ---------- -----
ATMPK5__B         ---------- ---------- -----
Cqu_c17078_1_g0   ---------- ---------- -----
ATMPK1__C         ---------- ---------- -----
Cqu_c02858_1_g0   ---------- ---------- -----
ATMPK7__C         ---------- ---------- -----
ATMPK2__C         ---------- ---------- -----
ATMPK14__C        ---------- ---------- -----
ATMPK8__D         ---------- ---------- -----
Cqu_c08055_1_g0   ---------- ---------- -----
ATMPK15__D        ---------- ---------- -----
ATMPK9__D         ---------- ---------- -----
ATMPK18__D        AHRNIGTISY -SAAS----- -----
ATMPK20__D        AHRKVGAVRY -GMSKMY--- -----
ATMPK19__D        AHRNIGAVGY -GMS------ -----
Cqu_c06169_1_g0   AHRNVGTVQY -GMS------ -----
Cqu_c03735_1_g0   AKSEVDDSSY LSLSRCVKRL MKYKE
ATMPK16__D        ---------- ---------- -----
Cqu_c07086_1_g0   ---------- ---------- -----
Cqu_c09059_1_g0   AHRNVGTVQY -GMS------ -----
ATMPK17__D        ---------- ---------- -----
Cqu_c04094_1_g0   ---------- ---------- -----
Cqu_c01012_1_g0   ---------- ---------- -----
Cqu_c14619_1_g0   -------QTR LPIHSLLGRL WLRTG
Cqu_c03126_1_g0   ---------- ---------- -----
Cqu_c01012_1_g0   ---------- ---------- -----
Consistency       0000000000 0000000000 00000
```

Additional supplemental data

GyrA, CheA sequences of quinoa endophytic bacteria

```
>GyrA partial sequence
AGCGTTATCGTATCCCGGGCGCTTCCGGATGTGCGTGACGGTCTGAAGCCGGTTCACAGA
CGGATTTTGTACGCGATGAATGATTTAGGCATGACCAGTGACAAACCATATAAAAAATCT
GCCCGTATCGTCGGTGAAGTTATCGGTAAGTACCACCCGCACGGTGACTCAGCGGTTTAC
GAATCAATGGTCAGAATGGCGCAGGATTTTAACAACCGCTACATGCTTGTTGACGGACAC
GGCAACTTCGGTTCGGTTGACGGCGACTCAGCGGCCGCGATGCGTTACACAGAAGCGAGA
ATGTCAAAAATCGCAATGGAAATCCTCCGGGACATTACGAAAGATACGATTGATTATCAA
GATAACTATGACGGCGCAGAAAGAGAACCTGTCGTCATGCCTTCGAGATTTCCGAATCTG
CTCGTCAACGGAGCTGCCGGTATCGCGGCCGGAATGGCGACAAATATTCCTCCGCATCAG
CTTGGGGAAGTCATTGAAGGGCGTGCTTGCCGTAAG

>CheA partial sequence
GGAAGGAGATGGTGCAGTCCATTATTGACGGAGGAGACGGCAAACGTGATATTTCTGAAG
TGAGTGCGAAGCTCGACGTAAATGCGGTGCATGAGACTGCGGCTTCAGCCGAAACAGCAG
AACCGCCGGCTTCAAAACAACAGGCTTCAACTGAATGGAATTATGATGAGTTCGAACGGA
CTGTTATTGAAGAGGCGGAAGAGCAAGGATTCAGCCGCTATGAAATAACGGTTTCCCTGA
ATGAAAGCTGCATGCTCAAAGCCGTGCGCGTGTACATGATATTTGAAAAGCTGAATGAAG
CCGGGGAAGTCGCAAAAACGATACCCGCCGCGGAAGTGCTTGAGACGGAAGATTTCGGAA
CGGATTTTCAGGTGTGTTTCTTAACAAAGC
```

List of primers used in colony PCRs

Flg_fo	ATGAGAATTAACCACAATAT	(Asano et al. 2001)
Flg_re	GTTGGTTTGCTTGAGCAAGC	
PeBA1_fo	ATGGCTAACCAGAAGAAGAAAAC	(Wang et al. 2016).
PeBA1_re	TTATCCGTTTACGATGGTGC	
BmyD_fo	GCTCTTGATGACCTTCTCTGCG	(Xu et al. 2013)
BmyD_re	ATTTTGTGCGATTGGTTCTGTT	
GyrA_fo	CAGTCAGGAAATGCGTACGTCCTT	(Reva et al. 2004)
GyrA_re	CAAGGTAATGCTCCAGGCATTGCT	
CheA_fo	AGGGCTGCCCATACATTAAAAGGAATGAGC	
CheA_re	AGTTTCAACCGGGACCATTCTCATATTCAG	

Asano Y, Onishi H, Tajima K, Shinozawa T (2001) Flagellin as a biomarker for Bacillus subtilis strains; Application to the DB9011 strain and the study of interspecific diversity in amino-acid sequences. Biosci Biotech Bioch 65: 1218-1222. doi: DOI 10.1271/bbb.65.1218.

Reva ON, Dixelius C, Meijer J, Priest FG (2004) Taxonomic characterization and plant colonizing abilities of some bacteria related to Bacillus amyloliquefaciens and Bacillus subtilis. FEMS microbiology ecology 48: 249-259. doi: 10.1016/j.femsec.2004.02.003.

Wang N, Liu M, Guo L, Yang X, Qiu D (2016) A Novel Protein Elicitor (PeBA1) from Bacillus amyloliquefaciens NC6 Induces Systemic Resistance in Tobacco. International Journal of Biological Sciences 12: 757-767. doi: 10.7150/ijbs.14333.

Xu Z, Shao J, Li B, Yan X, Shen Q, Zhang R (2013) Contribution of bacillomycin D in Bacillus amyloliquefaciens SQR9 to antifungal activity and biofilm formation. Applied and environmental microbiology 79: 808-815. doi: 10.1128/AEM.02645-12.

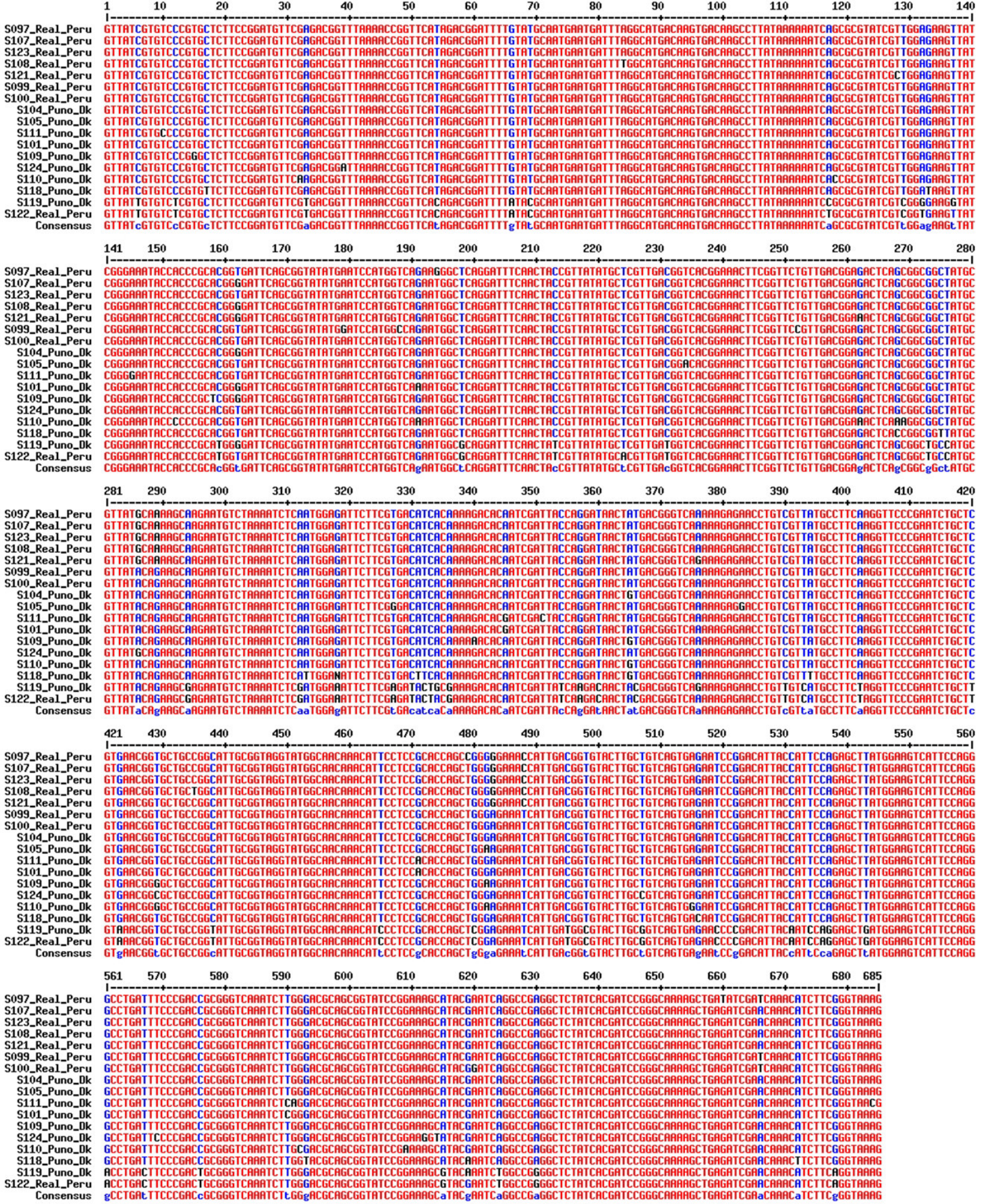

Suppl. figure S3

GyrA sequence alignment of bacteria from quinoa cultivars Puno (Denmark) and Real (Peru) See also phylogenetic tree in figure 2C. Metagenomic DNA from seeds served as template for PCR cloning.
